# Systematic Analysis of Metabolic Bottlenecks in the Methylerythritol 4-Phosphate (MEP) Pathway of Zymomonas mobilis

**DOI:** 10.1128/msystems.00092-23

**Published:** 2023-03-30

**Authors:** Daven B. Khana, Mehmet Tatli, Julio Rivera Vazquez, Sarathi M. Weraduwage, Noah Stern, Alexander S. Hebert, Edna Angelica Trujillo, David M. Stevenson, Joshua J. Coon, Thomas D. Sharky, Daniel Amador-Noguez

**Affiliations:** a DOE Great Lakes Bioenergy Research Center, University of Wisconsin-Madison, Madison, Wisconsin, USA; b Department of Bacteriology, University of Wisconsin-Madison, Madison, Wisconsin, USA; c Microbiology Doctoral Training Program, University of Wisconsin-Madison, Wisconsin, USA; d MSU-DOE Plant Research Laboratory, Michigan State University, East Lansing, Michigan, USA; e Genome Center of Wisconsin, Madison, Wisconsin, USA; f Department of Biomolecular Chemistry, University of Wisconsin-Madison, Madison, Wisconsin, USA; g Department of Chemistry, University of Wisconsin-Madison, Madison, Wisconsin, USA; h Morgridge Institute for Research, Madison, Wisconsin, USA; MS Bioscience

**Keywords:** *Zymomonas mobilis*, MEP pathway, metabolomics, metabolic bottleneck, isoprene, isoprenoid synthesis, mass spectrometry, isoprenoid pathway

## Abstract

Zymomonas mobilis is an industrially relevant aerotolerant anaerobic bacterium that can convert up to 96% of consumed glucose to ethanol. This highly catabolic metabolism could be leveraged to produce isoprenoid-based bioproducts via the methylerythritol 4-phosphate (MEP) pathway, but we currently have limited knowledge concerning the metabolic constraints of this pathway in Z. mobilis. Here, we performed an initial investigation of the metabolic bottlenecks within the MEP pathway of Z. mobilis using enzyme overexpression strains and quantitative metabolomics. Our analysis revealed that 1-deoxy-d-xylulose 5-phosphate synthase (DXS) represents the first enzymatic bottleneck in the Z. mobilis MEP pathway. DXS overexpression triggered large increases in the intracellular levels of the first five MEP pathway intermediates, of which the buildup in 2-C-methyl-d-erythritol 2,4-cyclodiphosphate (MEcDP) was the most substantial. The combined overexpression of DXS, 4-hydroxy-3-methylbut-2-enyl diphosphate (HMBDP) synthase (IspG), and HMBDP reductase (IspH) mitigated the bottleneck at MEcDP and mobilized carbon to downstream MEP pathway intermediates, indicating that IspG and IspH activity become the primary pathway constraints during DXS overexpression. Finally, we overexpressed DXS with other native MEP enzymes and a heterologous isoprene synthase and showed that isoprene can be used as a carbon sink in the Z. mobilis MEP pathway. By revealing key bottlenecks within the MEP pathway of Z. mobilis, this study will aid future engineering efforts aimed at developing this bacterium for industrial isoprenoid production.

**IMPORTANCE** Engineered microorganisms have the potential to convert renewable substrates into biofuels and valuable bioproducts, which offers an environmentally sustainable alternative to fossil-fuel-derived products. Isoprenoids are a diverse class of biologically derived compounds that have commercial applications as various commodity chemicals, including biofuels and biofuel precursor molecules. Thus, isoprenoids represent a desirable target for large-scale microbial generation. However, our ability to engineer microbes for the industrial production of isoprenoid-derived bioproducts is limited by an incomplete understanding of the bottlenecks in the biosynthetic pathway responsible for isoprenoid precursor generation. In this study, we combined genetic engineering with quantitative analyses of metabolism to examine the capabilities and constraints of the isoprenoid biosynthetic pathway in the industrially relevant microbe Zymomonas mobilis. Our integrated and systematic approach identified multiple enzymes whose overexpression in Z. mobilis results in an increased production of isoprenoid precursor molecules and mitigation of metabolic bottlenecks.

## INTRODUCTION

Metabolic engineering and synthetic biology are powerful tools capable of leveraging biological systems for the generation of valuable bioproducts, including biofuels (1, 2). One promising strategy to produce industrial quantities of bioproducts involves the microbial conversion of renewable feedstocks, such as lignocellulosic plant biomass (3, 4). Zymomonas mobilis is an aerotolerant anaerobic bacterium that possesses several desirable characteristics for industrial biofuel production. It is resistant against inhibitors present in lignocellulosic hydrolysates, has low biomass generation, and is a natural ethanologen that can rapidly convert up to 96% of consumed glucose to ethanol (5–10). Thus, the high catabolic rate at which Z. mobilis converts glucose to ethanol could be redirected toward the production of advanced biofuels (i.e., isobutanol) or other bioproducts, such as isoprenoids (9, 11–14).

**FIG 1:**
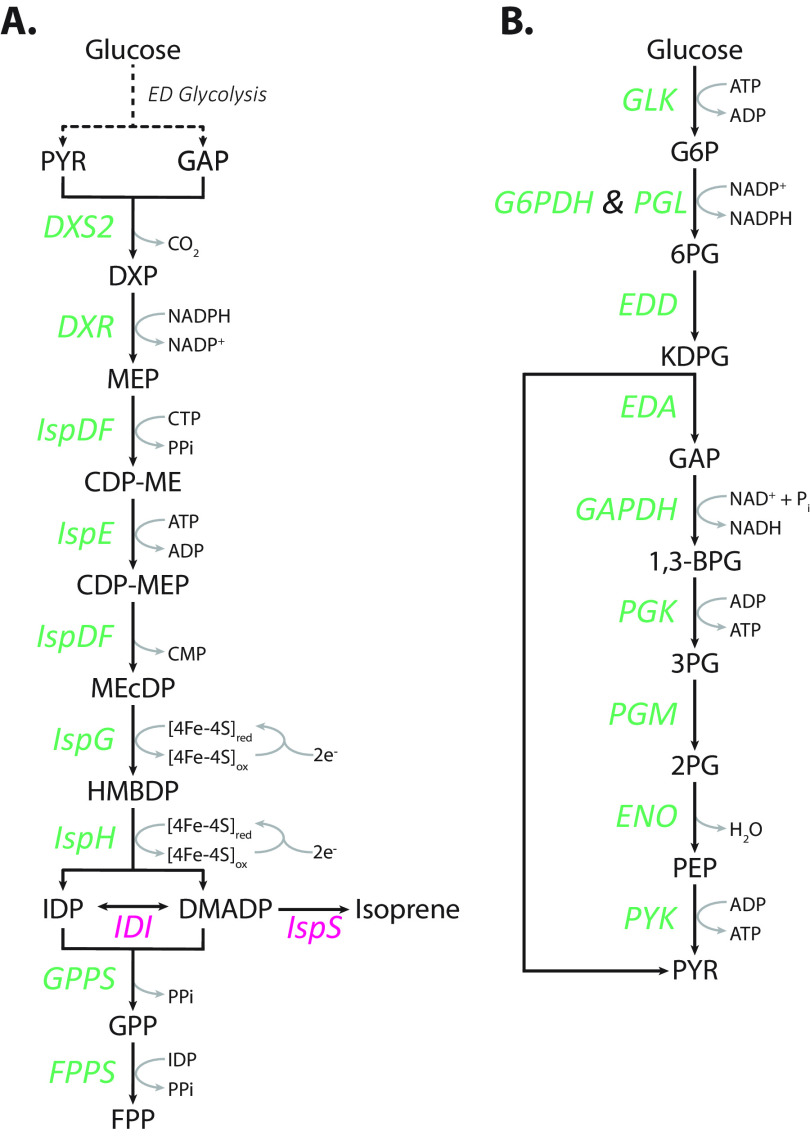
Z. mobilis generates the isoprenoid precursor molecules IDP and DMADP via the MEP pathway (A) from the Entner-Doudoroff (ED) glycolysis (B) intermediates glyceraldehyde 3-phosphate and pyruvate. Enzymes in green are native to Z. mobilis, and enzymes in pink were heterologously expressed for the purpose of this study. PYR, pyruvate; GAP, glyceraldehyde 3-phosphate; DXP, 1-deoxy-d-xylulose 5-phosphate; MEP, 2-C-methyl-d-erythritol 4-phosphate; CDP-ME, 4-diphosphocytidyl-2-C-methyl-d-erythritol; CDP-MEP, 4-diphosphocytidyl-2-C-methyl-d-erythritol 2-phosphate; MEcDP, 2-C-methyl-d-erythritol 2,4-cyclodiphosphate; HMBDP, 4-hydroxy-3-methylbut-2-enyl-diphosphate; IDP, isopentenyl diphosphate; DMADP, dimethylallyl diphosphate; GPP, geranyl pyrophosphate; FPP, farnesyl pyrophosphate; G6P, glucose 6-phosphate; 6PG, 6-phosphogluconate; KDPG, 2-keto-3-deoxy-6-phosphogluconate; 1,3-BPG, 1,3-bisphosphoglycerate; 3PG, 3-phosphoglycerate; 2PG, 2-phosphoglycerate; PEP, phosphoenolpyruvate; DXS2, DXP synthase; DXR, DXP reductoisomerase; IspDF, bifunctional enzyme MEP cytidyl transferase/MEcDP synthase; IspE, CDP-ME kinase; IspG, HMBDP synthase; IspH, HMBDP reductase; GPPS, GPP synthase; FPPS, FPP synthase; IDI, IDP isomerase; IspS, isoprene synthase; GLK, glucokinase; G6PDH, G6P dehydrogenase; PGL, 6-phosphogluconolactonase; EDD, 6PG dehydratase; EDA, KDPG aldolase; GAPDH, GAP dehydrogenase; PGK, phosphoglycerate kinase; PGM, phosphoglycerate mutase; ENO, enolase; PYK, PYR kinase.

Isoprenoids are a large and structurally diverse class of biologically derived compounds that are involved in numerous metabolic and regulatory activities (15–17). In addition to their native physiological roles, isoprenoids have commercial applications as pharmaceuticals, agrochemicals, pigments, and fragrances and have the potential to be used as biofuels or biofuel precursors (18–22). Isoprenoids are synthesized from the precursor molecules isopentenyl diphosphate (IDP) and its isomer dimethylallyl diphosphate (DMADP). IDP and DMADP can be produced via two distinct pathways: the mevalonate (MVA) pathway and the methylerythritol 4-phosphate (MEP) pathway. The MVA pathway is present in mammals, fungi, plant cytoplasm, archaea, and some Gram-positive bacteria, while the MEP pathway is present in green algae, plant chloroplasts, and most prokaryotes, including Z. mobilis (21, 23–25). The MEP pathway consists of seven enzymatic steps that generate IDP and DMADP from the glycolytic intermediates glyceraldehyde 3-phosphate (GAP) and pyruvate (Fig. 1A).

There are several traits that render Z. mobilis a viable metabolic engineering candidate for large-scale isoprenoid production. Its exclusive use of the highly thermodynamically favorable Entner-Doudoroff (ED) glycolytic pathway (Fig. 1B) generates the MEP pathway precursors pyruvate and GAP at high intracellular concentrations (Table 1) (10) and in fewer reaction steps than the classical Embden-Meyerhof-Parnas (EMP) glycolytic pathway, which altogether should facilitate high carbon flux from glucose to the MEP pathway. In addition, the high levels of hopanoids, a subclass of isoprenoids, in the lipid membrane of Z. mobilis indicates a MEP pathway with high native activity (26). Despite these advantages, there is little known about the metabolic regulation of the MEP pathway in Z. mobilis, which hinders engineering of Z. mobilis for large-scale isoprenoid production.

**TABLE 1:** Intracellular metabolite concentrations in Z. mobilis ZM4

Metabolite	Abbreviation	Molarity (M)	Source or reference
Glucose 6-phosphate	G6P	5.00E-03	10
6-Phosphogluconate	6PG	2.09E-03	10
2-Keto-3-deoxy-6-phosphogluconate	KDPG	6.39E-03	10
Glyceraldehyde 3-phosphate	GAP	1.00E-03	This study
Pyruvate	PYR	6.35E-03	This study
3-Phosphoglycerate	3PG	3.85E-03	10
Phosphoenolpyruvate	PEP	4.02E-05	10
1-Deoxy-d-xylulose 5-phosphate	DXP	1.68E-04	This study
2-C-methyl-d-erythritol 4-phosphate	MEP	1.40E-05	This study
4-Diphosphocytidyl-2-C-methyl-d-erythritol	CDP-ME	3.60E-05	This study
2-C-methyl-d-erythritol 2,4-cylcodiphosphate	MEcDP	1.25E-04	This study
4-Hydroxy-3-methylbut-2-enyl-diphosphate	HMBDP	7.40E-06	This study
Isopentenyl diphosphate-dimethylallyl diphosphate	IDP/ DMADP	7.09E-06	This study
Geranyl pyrophosphate	GPP	3.28E-06	This study
Farnesyl pyrophosphate	FPP	2.56E-05	This study
Adenosine triphosphate	ATP	2.81E-03	This study
Adenosine diphosphate	ADP	1.27E-03	This study
Adenosine monophosphate	AMP	8.89E-05	10
Guanosine triphosphate	GTP	1.72E-03	10
Guanosine diphosphate	GDP	4.23E-04	10
Guanosine monophosphate	GMP	5.16E-05	10
Uridine triphosphate	UTP	1.81E-03	10
Uridine diphosphate	UDP	4.47E-04	10
Uridine monophosphate	UMP	8.94E-05	10
Cytidine triphosphate	CTP	1.12E-03	This study
Cytidine monophosphate	CMP	3.42E-05	This study
Nicotinamide adenine dinucleotide (reduced)	NADH	4.13E-04	10
Nicotinamide adenine dinucleotide (oxidized)	NAD^+^	2.21E-03	10
Nicotinamide adenine dinucleotide phosphate (reduced)	NADPH	5.51E-04	10
Nicotinamide adenine dinucleotide phosphate (oxidized)	NADP^+^	3.00E-04	10

Previous studies in the bacteria Escherichia coli, Bacillus subtilis, and Rhodobacter sphaeroides and in the plant Arabidopsis thaliana have demonstrated that the individual or combinatorial overexpression of MEP enzymes are viable approaches to increase MEP pathway activity and offer insight behind the regulation of this pathway (27–30). In this study, we overexpressed MEP pathway enzymes individually and in various combinations and applied a quantitative metabolomics approach to investigate the capabilities and constraints of the Z. mobilis MEP pathway. We identified key bottlenecks that constitute primary targets for future efforts to engineer this bacterium for isoprenoid production.

## RESULTS

### Quantitation of intracellular MEP pathway intermediates in Z. mobilis.

**FIG 2:**
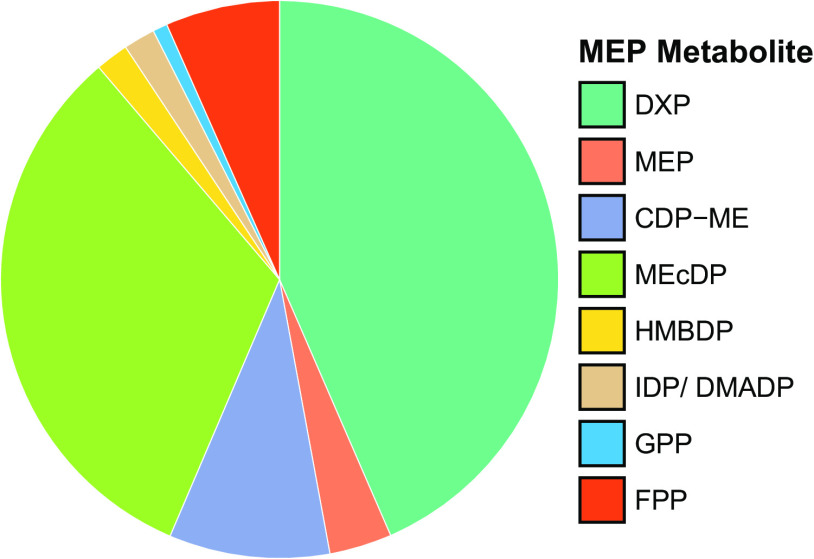
Relative levels of MEP pathway intermediates and downstream metabolites GPP and FPP. DXP and MEcDP are the most abundant MEP pathway metabolites in wild-type Z. mobilis. Intracellular metabolite concentrations were measured using an isotope ratio-based approach (see Materials and Methods). Data represent averages of six biological replicates. See Table 1 for the absolute metabolite concentrations of these metabolites. DXP, 1-deoxy-d-xylulose 5-phosphate; MEP, 2-C-methyl-d-erythritol 4-phosphate; CDP-ME, 4-diphosphocytidyl-2-C-methyl-d-erythritol; MEcDP, 2-C-methyl-d-erythritol 2,4-cyclodiphosphate; HMBDP, 4-hydroxy-3-methylbut-2-enyl diphosphate; IDP, isopentenyl diphosphate; DMADP, dimethylallyl diphosphate; GPP, geranyl pyrophosphate; FPP, farnesyl pyrophosphate.

To begin our investigation of the MEP pathway in Z. mobilis, we used a liquid chromatography-mass spectrometry (LC-MS) isotope ratio-based approach (31) to measure the intracellular concentrations of seven of the eight MEP pathway intermediates in Z. mobilis ZM4 (ATCC 31821) grown anaerobically in minimal media. The intermediates included 1-deoxy-d-xylulose 5-phosphate (DXP), 2-C-methyl-d-erythritol 4-phosphate (MEP), 4-diphosphocytidyl-2-C-methyl-d-erythritol (CDP-ME), 2-C-methyl-d-erythritol 2,4-cyclodiphosphate (MEcDP), 4-hydroxy-3-methylbut-2-enyl diphosphate (HMBDP), isopentenyl diphosphate (IDP), and dimethylallyl diphosphate (DMADP). We also measured energy cofactors (i.e., ATP, ADP, CTP, and CMP), and the downstream isoprenoids geranyl pyrophosphate (GPP) and farnesyl pyrophosphate (FPP). The MEP pathway intermediate 4-diphosphocytidyl-2-C-methyl-d-erythritol 2-phosphate (CDP-MEP) could not be quantified due to the lack of a purified standard, but changes in its relative levels across experiments were still obtained. Our analysis revealed that DXP and MEcDP are natively the most abundant intermediates in the MEP pathway and are >17-fold higher than the least abundant MEP intermediates HMBDP and IDP/DMADP (the isomers IDP and DMADP were measured as a combined pool, see Materials and Methods) (Fig. 2; Table 1; see Table S1 in the supplemental material). GAP and pyruvate concentrations are 6- and 38-fold higher, respectively, than the first MEP pathway intermediate DXP, suggesting that the first reaction in the MEP pathway of Z. mobilis is highly forward driven. Finally, we observed that the combined pool of MEP intermediates (excluding cofactors and nucleotide triphosphates) is less than 1/50th the size of the pool of glycolytic intermediates (10).

10.1128/msystems.00092-23.7 TABLE S1 Intracellular metabolite concentrations in Z. mobilis ZM4 with 95% confidence intervals. Download Table S1, DOCX file, 0.02 MB. Copyright © 2023 Khana et al. 2023 Khana et al. https://creativecommons.org/licenses/by/4.0/ This content is distributed under the terms of the Creative Commons Attribution 4.0 International license.

### Individual overexpression of MEP pathway enzymes results in large changes to the levels of MEP pathway intermediates.

To identify bottlenecks and investigate carbon flow through the MEP pathway in Z. mobilis, we overexpressed MEP pathway enzymes individually (i.e., DXS2, DXP synthase; DXR, DXP reductoisomerase; IspDF, bifunctional enzyme MEP cytidyl transferase/MEcDP synthase; IspE, CDP-ME kinase; IspG, HMBDP synthase; and IspH, HMBDP reductase) and monitored changes in the levels of metabolic intermediates from the MEP pathway and primary metabolism (e.g., ED glycolysis, nucleotides, and cofactors) via LC-MS metabolomics. To generate the individual overexpression strains, MEP genes cloned from Z. mobilis were inserted into the isopropyl β-d-1-thiogalactopyranoside (IPTG)-inducible plasmid pRL814 (32) and conjugated into Z. mobilis (see Materials and Methods; see Table S2 in the supplemental material). MEP enzyme overexpression strains were grown anaerobically in defined minimal media to an optical density at 600 nm (OD_600_) of 0.2 before induction with IPTG. Intracellular metabolites and samples for proteomics analyses were collected at mid-log phase (OD_600_, 0.5), and changes in metabolite and protein levels were measured against Z. mobilis harboring green fluorescent protein (GFP) in pRL814. To confirm the overexpression of target proteins in the individual overexpression strains, we performed label-free quantitative (LFQ) proteomics analyses via LC-tandem MS (LC-MS/MS). Protein levels for DXS2, DXR, IspDF, IspE, and IspG increased 32- to 92-fold over basal levels, while the overexpression of IspH led to a lesser increase of 5-fold (see Fig. S1A in the supplemental material). We observed no major changes to the expression of other cellular proteins in these strains, including enzymes in the MEP pathway or ED glycolysis.

10.1128/msystems.00092-23.1 FIG S1 Log_2_ fold changes in MEP pathway and ED glycolytic enzymes were measured postoverexpression of MEP pathway enzymes (A) and MEP pathway enzymes with heterologous proteins (B). Protein levels were measured against Z. mobilis overexpressing GFP or against background signal (i.e., IDI and IspS). Red, white, and green indicate increased, no change, and decreased protein expression, respectively. ND and NA indicate nondetectable and enzymes omitted from analysis, respectively. Data for the DXS2, DXR, IspDF, and IspE overexpression strains represent one biological replicate, and data for the IspG, IspH, DXS2_IspS, and DXS2_IspS_IDI overexpression strains represent three biological replicates. Abbreviations: DXS2, DXP synthase; DXR, DXP reductoisomerase; IspDF, bifunctional enzyme MEP cytidyl transferase/MEcDP synthase; IspE, CDP-ME kinase; IspG, HMBDP synthase; IspH, HMBDP reductase; Fpr, flavodoxin/ferredoxin NADP^+^ reductase; FldA, Flavodoxin I; FdxA, Ferredoxin; IDI, IDP isomerase; IspS, isoprene synthase; GLF, glucose facilitated diffusion protein; GLK, glucokinase; G6PDH, G6P dehydrogenase; PGL, 6-phosphogluconolactonase; EDD, 6PG dehydratase; EDA, KDPG aldolase; GAPDH, GAP dehydrogenase; PGK, phosphoglycerate kinase; PGM, phosphoglycerate mutase; ENO, enolase; PYK, PYR kinase. Download FIG S1, PDF file, 0.5 MB. Copyright © 2023 Khana et al. 2023 Khana et al. https://creativecommons.org/licenses/by/4.0/ This content is distributed under the terms of the Creative Commons Attribution 4.0 International license.

10.1128/msystems.00092-23.8 TABLE S2 List of engineered Z. mobilis strains constructed for this study. Download Table S2, DOCX file, 0.02 MB. Copyright © 2023 Khana et al. 2023 Khana et al. https://creativecommons.org/licenses/by/4.0/ This content is distributed under the terms of the Creative Commons Attribution 4.0 International license.

**FIG 3:**
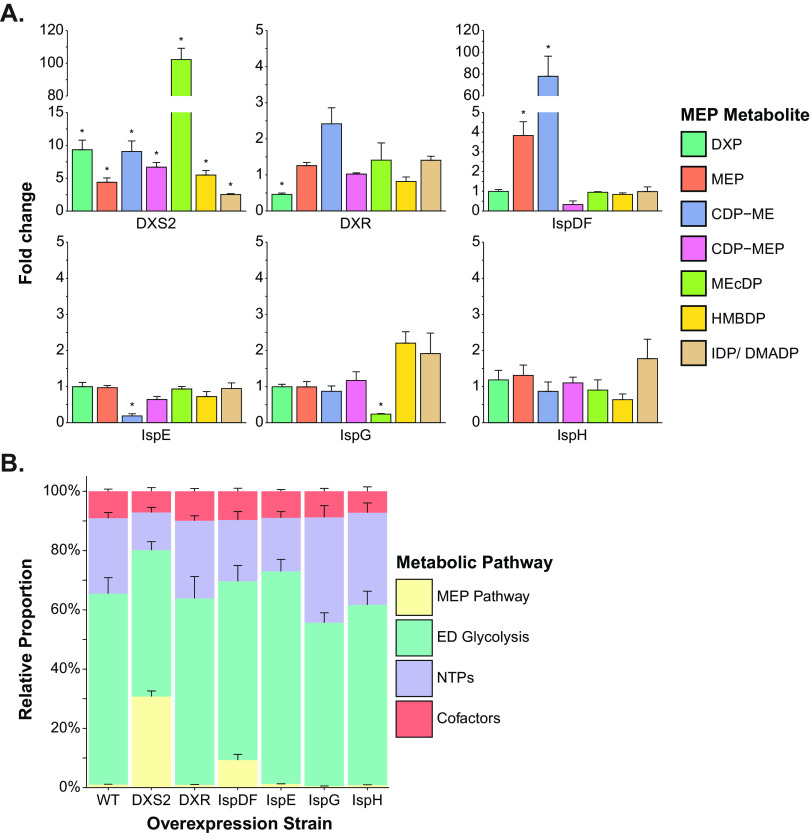
The overexpression of MEP enzymes can elicit major changes to the levels of MEP pathway metabolites. (A) Relative changes to the intracellular levels of MEP pathway intermediates postoverexpression of the MEP enzymes DXS2, DXR, IspDF, IspE, IspG, and IspH were measured relative to Z. mobilis overexpressing GFP. (B) The individual overexpression of DXS2 and IspDF in Z. mobilis results in large carbon accumulation within the MEP pathway. Each category (MEP pathway, yellow; ED glycolysis, green; nucleotide triphosphates, purple; and cofactors, orange) represents the sum of all individual absolute metabolite concentrations that belong to that group. Data represent averages of three biological replicates. Error bars show ± standard deviation. Some error bars are too small to be visible in this representation. Asterisks indicate statistical significance (FDR, <0.05). DXP, 1-deoxy-d-xylulose 5-phosphate; MEP, 2-C-methyl-d-erythritol 4-phosphate; CDP-ME, 4-diphosphocytidyl-2-C-methyl-d-erythritol; CDP-MEP, 4-diphosphocytidyl-2-C-methyl-d-erythritol 2-phosphate; MEcDP, 2-C-methyl-d-erythritol 2,4-cyclodiphosphate; HMBDP, 4-hydroxy-3-methylbut-2-enyl diphosphate; IDP, isopentenyl diphosphate; DMADP, dimethylallyl diphosphate; WT, wild type; DXS2, DXP synthase; DXR, DXP reductoisomerase; IspDF, bifunctional enzyme MEP cytidyl transferase/MEcDP synthase; IspE, CDP-ME kinase; IspG, HMBDP synthase; IspH, HMBDP reductase.

We observed that the individual overexpression of some MEP enzymes resulted in large changes to the levels of MEP pathway intermediates (Fig. 3). Most notably, the overexpression of DXS2 led to significant increases to all MEP pathway intermediates, with the most substantial increases occurring in DXP (9.3-fold), CDP-ME (9.1-fold), and MEcDP (102-fold) levels, which suggests that DXS2 is a rate-limiting step in the MEP pathway of Z. mobilis (Fig. 3A). Interestingly, the buildup of MEcDP translated only to relatively minor increases in the downstream MEP metabolites HMBDP and IDP/DMADP (5.5- and 2.5-fold increase, respectively), suggesting a bottleneck at IspG. In contrast, when DXR was overexpressed, we measured only a modest decrease in DXP levels (0.46-fold) and no significant changes to the levels of downstream intermediates (Fig. 3A). These moderate responses in metabolite levels suggest that DXR alone is not a major rate-limiting step in Z. mobilis. Similar to other bacteria, the IspD and IspF activities are performed by the single bifunctional enzyme IspDF in Z. mobilis (33–35). The overexpression of IspDF resulted in a 78-fold increase in CDP-ME levels and an unexpected 3.8-fold increase in MEP levels but no significant changes in other MEP metabolites (Fig. 3A). These results suggest that the IspD domain of the IspDF enzyme complex is potentially more active than the IspF component in Z. mobilis and that IspDF is potentially another rate-limiting step in the MEP pathway. When IspE was overexpressed in Z. mobilis, we observed a significant reduction in CDP-ME levels (0.19-fold) but no significant changes in any other MEP pathway intermediate (Fig. 3A). Continuing to the next enzyme in the pathway, the overexpression of IspG led to a significant decrease in the levels of its substrate MEcDP (0.24-fold). We also observed some notable trends in other metabolites, such as a small increase in the IspG product HMBDP (2.2-fold) and the downstream intermediates IDP/DMADP (1.9-fold) (Fig. 3A). Similarly, while the overexpression of IspH induced no significant metabolite changes, notable trends included a minor reduction of 0.64-fold in HMBDP levels and a slight increase of 1.8-fold in IDP/DMADP levels (Fig. 3A).

The individual overexpression of DXS2 and IspDF resulted in large increases to the combined pool of MEP pathway intermediates (Fig. 3B). For DXS2, the combined pool of the absolute intracellular concentrations of MEP pathway intermediates (excluding cofactors and nucleotide triphosphates) increased by 39-fold, approaching >60% the pool size of ED pathway intermediates, which are among the most abundant metabolites in Z. mobilis primary metabolism (10). Overall, these data suggest that DXS2 and IspDF overexpression can greatly increase carbon flow into the MEP pathway of Z. mobilis.

### Dynamic changes in the levels of MEP pathway intermediates during enzyme overexpression reveal metabolic bottlenecks.

**FIG 4:**
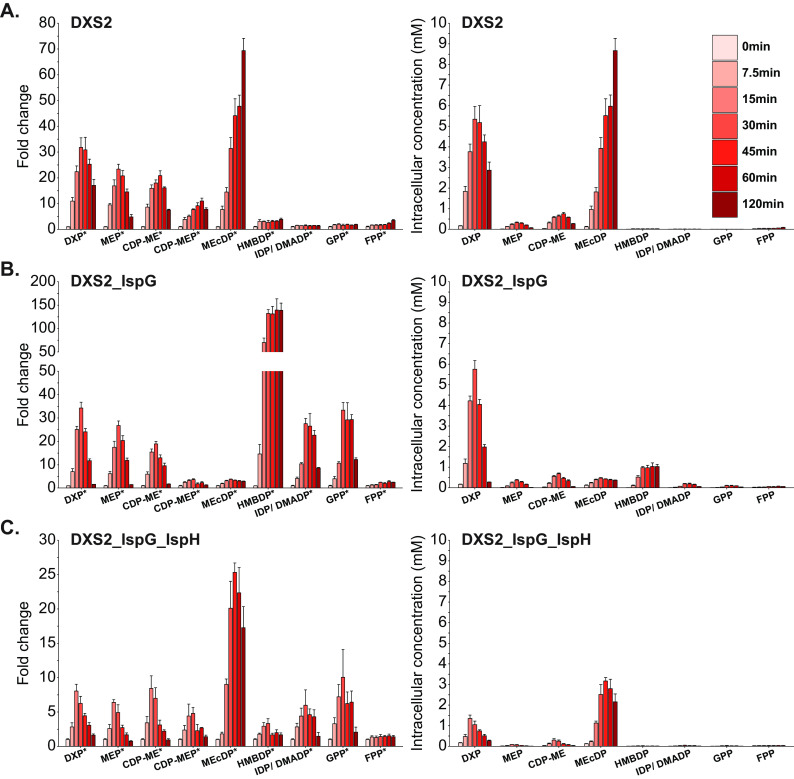
The combinatorial overexpression of DXS2 with other MEP enzymes alters metabolic bottlenecks present during individual DXS2 overexpression. Fold change data (left) relative to preinduction (time point 0) metabolite levels and absolute metabolite concentrations (right) for MEP metabolites at 7.5, 15, 30, 45, 60, and 120 min postoverexpression of DXS2 (A); DXS2 and IspG (B); and DXS2, IspG, and IspH (C). Data represent averages of three biological replicates. Error bars show ± standard deviation. Some error bars are too small to be visible in this representation. Asterisks located next to metabolite names on the X-axis indicate statistical significance of at least one datapoint within the time course for the indicated metabolite (FDR, <0.05). DXP, 1-deoxy-d-xylulose 5-phosphate; MEP, 2-C-methyl-d-erythritol 4-phosphate; CDP-ME, 4-diphosphocytidyl-2-C-methyl-d-erythritol; CDP-MEP, 4-diphosphocytidyl-2-C-methyl-d-erythritol 2-phosphate; MEcDP, 2-C-methyl-d-erythritol 2,4-cyclodiphosphate; HMBDP, 4-hydroxy-3-methylbut-2-enyl diphosphate; IDP, isopentenyl diphosphate; DMADP, dimethylallyl diphosphate; GPP, geranyl pyrophosphate; FPP, farnesyl pyrophosphate; DXS2, DXP synthase; IspG, HMBDP synthase; IspH, HMBDP reductase.

To continue our investigation of MEP pathway bottlenecks in Z. mobilis, we overexpressed DXS2 individually and in various combinations with other MEP enzymes (Table S2) and monitored dynamic changes in metabolism. Engineered strains were grown anaerobically in minimal media, and intracellular metabolites were extracted when cells reached an OD_600_ of 0.35, which represented the initial time point (0 min). Cultures were then induced with IPTG, and metabolites were collected at 7.5, 15, 30, 45, 60, and 120 min postinduction (Fig. 4).

DXS2 overexpression resulted in a rapid accumulation of MEP intermediates, from DXP to HMBDP, starting at 7.5 min postinduction (Fig. 4A). DXP, MEP, CDP-ME, and CDP-MEP reached maximum levels (32-, 23-, 21-, and 11-fold, respectively) between 30 and 45 min postinduction. By 120 min, these metabolites settled to levels similar to those observed during the single-time-point DXS2 overexpression experiment (Fig. 3A). However, MEcDP continued to accumulate up to 120 min postinduction, reaching a 69-fold increase, while the accumulation in downstream MEP intermediates remained relatively minor (<4-fold). Overall, the overexpression of DXS2 led to large accumulations in the intracellular concentrations of DXP and MEcDP (Fig. 4A). Thus, in agreement with our results from the single-time-point experiment (Fig. 3A), these time course data support the conclusion that DXS2 is a rate-limiting step and the first major enzymatic bottleneck in the MEP pathway of Z. mobilis. In addition, these observations also suggest that with heightened DXS2 activity, IspG and/or IspH become major bottlenecks in the MEP pathway, while DXR represents a less severe enzymatic constraint.

The combined overexpression of DXS2 and IspG triggered an accumulation of all MEP intermediates starting at 7.5 min postinduction (Fig. 4B). Critically, when IspG was overexpressed alongside DXS2, the large buildup of MEcDP observed during DXS2 overexpression did not occur, and instead, we observed a large increase in HMBDP, which accumulated by 140-fold at 60 min postinduction. This increase in HMBDP levels extended to the downstream intermediates IDP/DMADP and GPP, which accumulated by 28- and 33-fold, respectively, at 30 min postinduction. Despite the large fold change increase in HMBDP levels, the absolute intracellular concentration of this MEP pathway intermediate remained lower than that of DXP (Fig. 4B). Overall, the combinatorial overexpression of DXS2 and IspG effectively mobilized carbon from MEcDP to HMBDP and to a lesser extent IDP/DMADP, which indicates that IspG is a rate-limiting step in the MEP pathway during DXS2 overexpression. The accumulation in HMBDP levels observed during DXS2 and IspG activation also indicated that the next bottleneck in this strain was IspH. In alignment with our expectations, when we overexpressed IspH in combination with DXS2 and IspG, HMBDP levels accumulated only by 3.3-fold at 30 min postinduction (Fig. 4C). Interestingly, we observed an unexpected buildup of MEcDP (25-fold by 45 min) in this strain that was significantly higher than the one in the DXS2_IspG overexpression strain; however, this accumulation was transient and was lower than that during individual DXS2 activation (Fig. 3A). Notably, the absolute intracellular concentrations of DXP and MEcDP both decreased during the overexpression of DXS2, IspG, and IspH (Fig. 4C), indicating an effective mitigation in the MEcDP bottleneck. Overall, these data suggest that during DXS2 overexpression, IspG and IspH become major rate-limiting enzymes and that their overexpression can mitigate the accumulation of MEcDP observed during DXS2 activation.

### Glycolytic intermediates, nucleotide triphosphates, and cofactors remain largely unaffected by MEP pathway enzyme overexpression.

In addition to MEP pathway intermediates, we also monitored changes to central carbon metabolites, energy molecules, and redox cofactors during MEP enzyme overexpression (see Fig. S2 and S3 in the supplemental material). With few exceptions, ED glycolytic intermediates, nucleotide triphosphates (NTPs), and redox cofactors remained largely unaffected during MEP enzyme overexpression. For example, time course experiments revealed a slight reduction in 6-phosphogluconate (6PG) levels and a modest accumulation in phosphoenolpyruvate (PEP) during individual DXS2 overexpression (Fig. S3A). However, these trends were not maintained in the DXS2_IspG or DXS2_IspG_IspH overexpression strains (Fig. S3B and C). Other strains overexpressing individual MEP pathway enzymes (i.e., DXR, IspE, IspDF, IspG, and IspH) also did not display consistent changes to glycolytic intermediates, NTPs, or cofactors (Fig. S2).

10.1128/msystems.00092-23.2 FIG S2 Measurements in central carbon metabolites postoverexpression of individual MEP enzymes reveals no major alterations. Fold changes in ED glycolytic intermediates (A) and NTPs/cofactors (B) postoverexpression of MEP enzymes were measured relative to Z. mobilis overexpressing GFP. Data represent averages of three biological replicates. Error bars show ± standard deviation. Asterisks indicate statistical significance (FDR, <0.05). Abbreviations: G6P, glucose 6-phosphate; 6PG, 6-phosphogluconate; KDPG, 2-keto-3-deoxy-6-phosphogluconate; GAP, glyceraldehyde 3-phosphate; 1,3-BPG, 1,3-bisphosphoglycerate; 3PG, 3-phosphoglycerate; PEP, phosphoenolpyruvate; DXS2, DXP synthase; DXR, DXP reductoisomerase; IspDF, bifunctional enzyme MEP cytidyl transferase/MEcDP synthase; IspE, CDP-ME kinase; IspG, HMBDP synthase; IspH, HMBDP reductase. Download FIG S2, PDF file, 0.5 MB. Copyright © 2023 Khana et al. 2023 Khana et al. https://creativecommons.org/licenses/by/4.0/ This content is distributed under the terms of the Creative Commons Attribution 4.0 International license.

10.1128/msystems.00092-23.3 FIG S3 Fold changes relative to preinduction (timepoint 0) metabolite levels in ED glycolytic intermediates (left) and NTPs/cofactors (right) at 7.5, 15, 30, 45, 60, and 120 minutes postoverexpression of DXS2 (A); DXS2 and IspG (B); DXS2, IspG, and IspH (C); DXS2 and IspS (D); DXS2, IspS, and IDI (E); DXS2, IspG, IspH, and IspS (F); and DXS2, IspG, IspH, IDI, and IspS (G). Data represent averages of three biological replicates. Error bars show ± standard deviation. Some error bars are too small to be visible in this representation. Asterisks indicate statistical significance (FDR, <0.05). Datapoints represented with an “X” were undetectable. Abbreviations: G6P, glucose 6-phosphate; 6PG, 6-phosphogluconate; KDPG, 2-keto-3-deoxy-6-phosphogluconate; GAP, glyceraldehyde 3-phosphate; 1,3-BPG, 1,3-bisphosphoglycerate; 3PG, 3-phosphoglycerate; PEP, phosphoenolpyruvate; PYR, pyruvate; DXS2, DXP synthase; IspG, HMBDP synthase; IspH, HMBDP reductase; IspS, isoprene synthase; IDI, IDP isomerase. Download FIG S3, PDF file, 0.7 MB. Copyright © 2023 Khana et al. 2023 Khana et al. https://creativecommons.org/licenses/by/4.0/ This content is distributed under the terms of the Creative Commons Attribution 4.0 International license.

### Introducing a MEP pathway carbon sink via the heterologous expression of isoprene synthase.

**FIG 5:**
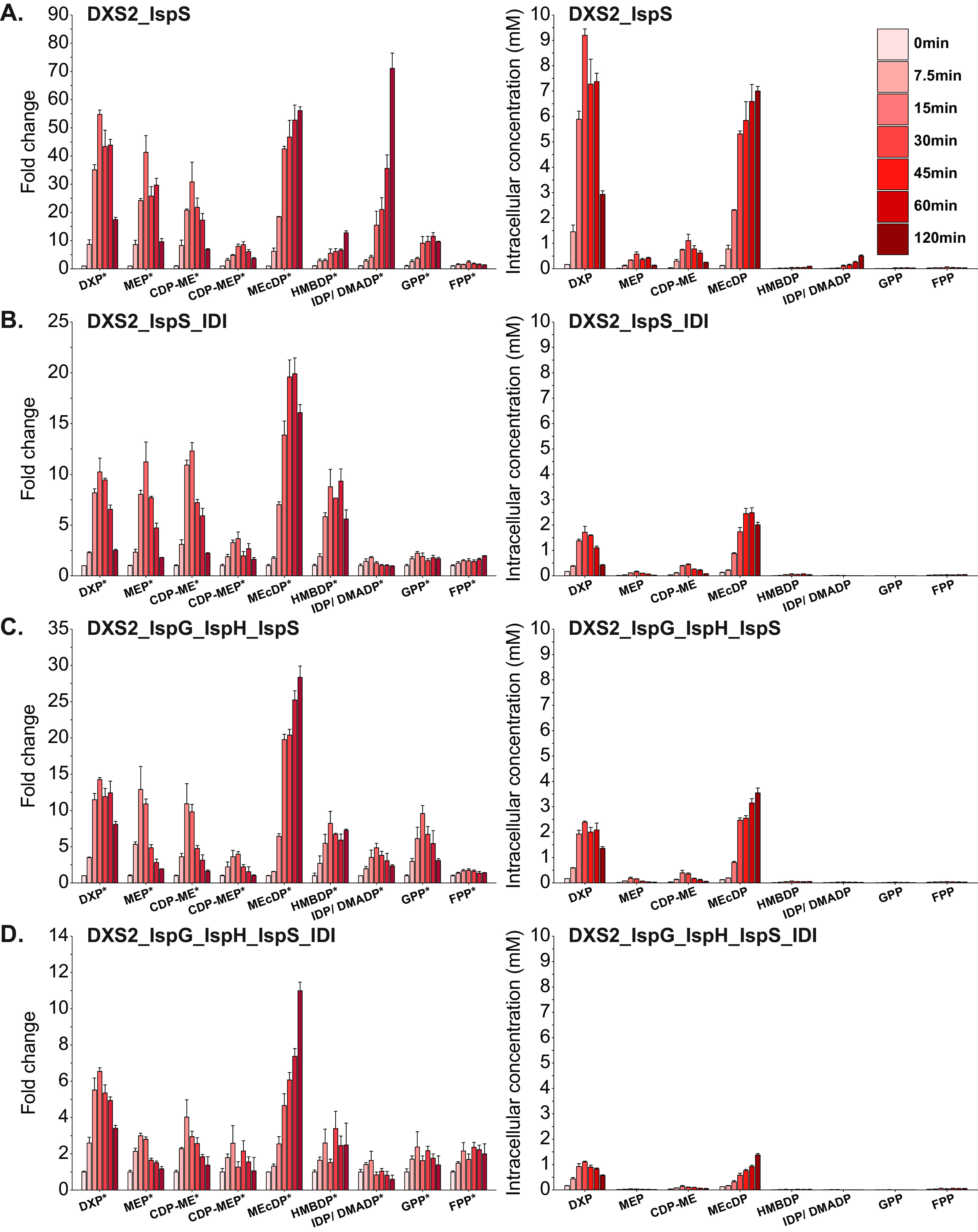
The overexpression of isoprene synthase in combination with other MEP pathway enzymes decreases carbon accumulation in the MEP pathway resulting from individual DXS2 overexpression. Fold change data (left) relative to preinduction (time point 0) metabolite levels and absolute metabolite concentrations (right) for MEP metabolites at 7.5, 15, 30, 45, 60, and 120 min postoverexpression of DXS2 and IspS (A); DXS2, IspS, and IDI (B); DXS2, IspG, IspH, and IspS (C); and DXS2, IspG, IspH, IDI, and IspS (D). Data represent averages of three biological replicates. Error bars show ± standard deviation. Some error bars are too small to be visible in this representation. Asterisks located next to metabolite names on the X-axis indicate statistical significance of at least one datapoint within the time course for the indicated metabolite (FDR, <0.05). DXP, 1-deoxy-d-xylulose 5-phosphate; MEP, 2-C-methyl-d-erythritol 4-phosphate; CDP-ME, 4-diphosphocytidyl-2-C-methyl-d-erythritol; CDP-MEP, 4-diphosphocytidyl-2-C-methyl-d-erythritol 2-phosphate; MEcDP, 2-C-methyl-d-erythritol 2,4-cyclodiphosphate; HMBDP, 4-hydroxy-3-methylbut-2-enyl diphosphate; IDP, isopentenyl diphosphate; DMADP, dimethylallyl diphosphate; GPP, geranyl pyrophosphate; FPP, farnesyl pyrophosphate; DXS2, DXP synthase; IspG, HMBDP synthase; IspH, HMBDP reductase; IDI, IDP isomerase; IspS, isoprene synthase.

To enhance carbon flow through the MEP pathway and to reduce the accumulation of DXP and MEcDP observed in the DXS2 and DXS2_IspG_IspH strains, we investigated the overexpression of isoprene synthase (IspS) (Table S2). Isoprene synthase dephosphorylates DMADP to generate pyrophosphate and isoprene (Fig. 1A), which is a highly volatile compound that readily diffuses out of the cell and growth media (36–39). Thus, isoprene can potentially act as a carbon sink in the MEP pathway and mobilize carbon away from upstream metabolites. Similar to the previous time course experiments, we overexpressed DXS2 and IspS with a combination of MEP enzymes and tracked dynamic changes in MEP pathway intermediates, central carbon metabolites, and cofactors at various time points post-enzyme overexpression (Fig. 5 and Fig. S3).

The combined overexpression of DXS2 and IspS led to similar changes in the levels of MEP pathway intermediates to those observed during individual DXS2 overexpression. Namely, we observed a large accumulation of DXP and MEcDP, with lesser accumulations in the other intermediates (Fig. 5A). Interestingly, we observed an unexpected accumulation in IDP/DMADP, which increased by 71-fold at 120 min postoverexpression. Despite this large fold change increase, however, the absolute intracellular levels of DXP and MEcDP remained far greater than IDP/DMADP levels, indicating that the severe bottleneck at IspG in the DXS2 overexpression strain was still present in the DXS2_IspS strain. Using an alternative chromatography method (see Materials and Methods), we separated the IDP/DMADP isomers and determined that during DXS2 and IspS overexpression, there was 25-fold more IDP than DMADP (see Fig. S4 in the supplemental material). This finding suggested that Z. mobilis cannot efficiently isomerize IDP to DMADP, which is consistent with the lack of genetic evidence for an endogenous IDP/DMADP isomerase (IDI) in this organism (40). Thus, we hypothesized that the heterologous expression of IDI (i.e., from E. coli) (Table S2) could mitigate the observed IDP accumulation. As expected, IDI overexpression with DXS2 and IspS did not result in an accumulation of IDP/DMADP, whose levels did not rise above 1.8-fold (Fig. 5B). Notably, the combined overexpression of DXS2, IspS, and IDI also substantially decreased the accumulation of DXP and MEcDP compared to combined DXS2 and IspS overexpression (Fig. 5B). While the reason for this result is unclear, it is plausible that IDI expression increases carbon flow toward DMADP and its downstream products, which in turn facilitates carbon flow from the upstream intermediates DXP and MEcDP.

10.1128/msystems.00092-23.4 FIG S4 LC-MS separation of the isomers IDP (pink) and DMADP (blue) in the DXS2_IspS strain reveals an imbalance in the IDP and DMADP ratio. Standards (35 μM) of IDP and DMADP (A) and metabolite extract from the DXS2_IspS overexpression strain (B) were aniline derivatized and analyzed via LC-MS using a 4.6- by 250-mm β-cyclodextrin chiral column with a 5.0-μm particle size (Astec Cyclobond I 2000) (see Materials and Methods). Due to the larger pore size of this column, it is less sensitive, and thus, the ability to separate IDP and DMADP was successful only in the DXS2_IspS, which produced the highest levels of these metabolites. Download FIG S4, PDF file, 0.4 MB. Copyright © 2023 Khana et al. 2023 Khana et al. https://creativecommons.org/licenses/by/4.0/ This content is distributed under the terms of the Creative Commons Attribution 4.0 International license.

We also overexpressed DXS2 and IspS together with IspG and IspH and found that the accumulation in DXP, MEcDP, and IDP/DMADP was lower than that observed during DXS2 and IspS overexpression, suggesting once again that IspG and IspH overexpression can effectively mitigate these metabolic bottlenecks (Fig. 5C). Finally, we overexpressed a combination of DXS2, IspG, IspH, IspS, and IDI, which resulted in the smallest accumulations in the intracellular concentrations of DXP, MEcDP, and IDP/DMADP across all four strains (Fig. 5D), suggesting that this combination of enzymes is capable of mitigating the bottlenecks common to all strains overexpressing DXS2.

Across the four IspS overexpression strains, we noticed some consistent trends in changes to glycolytic intermediates (Fig. S3), such as reductions in glucose 6-phosphate and 2-keto-3-deoxy-6-phosphogluconate (KDPG) levels. Despite the decrease in KDPG levels, we observed only minor reductions in GAP and pyruvate for the strains expressing IDI (Fig. S3E and G). However, we did not detect any discernible trends in changes to NTPs or cofactors in the IspS overexpression strains (Fig. S3D to G).

To investigate if the resolution of the metabolic bottlenecks at DXP and MEcDP observed in our combinatorial IspS overexpression strains translated to increased isoprene production, we monitored isoprene production using a fast isoprene sensor (FIS) (41–43). IspS overexpression strains were grown anaerobically in unsealed flasks and induced with IPTG at an OD_600_ of 0.35. Cultures were grown for an additional 3 h, at which point aliquots were transferred to sealed vials. Following 10 min of growth, the headspace of the sealed vial was sampled and injected into the FIS to measure gaseous isoprene levels (see Materials and Methods).

Background levels of isoprene production (5.6 nmol_isoprene_ · mmol_glucose_^−1^) in Z. mobilis overexpressing GFP were consistent with results from previous studies that established the spontaneous dephosphorylation of DMADP to isoprene (Table 2) (44). The heterologous overexpression of IspS alone in Z. mobilis did not have a significant effect on isoprene production, indicating that the sole overexpression of IspS is insufficient to redirect MEP pathway intermediates toward isoprene production. However, the combined overexpression of DXS2 and IspS significantly increased isoprene production by 5.9-fold compared to background levels to 33.3 nmol_isoprene_ · mmol_glucose_^−1^. Surprisingly, the additional overexpression of IDI in the DXS2_IspS_IDI strain decreased the isoprene production to 16.3 nmol_isoprene_ · mmol_glucose_^−1^. The combined overexpression of DXS2, IspG, IspH, and IspS or the combined overexpression of DXS2, IspG, IspH, IspS, and IDI resulted in only 18.1 and 20.1 nmol_isoprene_ · mmol_glucose_^−1^ isoprene production, respectively, which were both lower than the isoprene production in the DXS2_IspS overexpression strain (Table 2).

**TABLE 2:** Isoprene production in Z. mobilis strains overexpressing isoprene synthase

Strain name	Overexpressing gene(s)	Isoprene production (nmol_isoprene_ mmol_glucose_^−1^)^*a*^
ZM4_GFP	GFP	5.6 ± 0.5 (*n* = 4)
ZM4_IspS	IspS	5.8 ± 2.9 (*n* = 3)
ZM4_DXS2_IspS	DXS2, IspS	33.3 ± 8.6^*b*^ (*n* = 5)
ZM4_DXS2_IspS_IDI	DXS2, IspS, IDI	16.3 ± 3.7 (*n* = 8)
ZM4_DXS2_IspG_IspH_IspS	DXS2, IspG, IspH, IspS	18.1 ± 4.0 (*n* = 5)
ZM4_DXS2_IspG_IspH_IspS_IDI	DXS2, IspG, IspH, IspS, IDI	20.1 ± 5.9^*b*^ (*n* = 5)

a Isoprene production represents the average of at least three biological replicates per strain ± standard error.

b Statistically significant difference in isoprene production compared to ZM4_GFP (*P* < 0.05).

## DISCUSSION

This study presents an initial investigation of metabolic bottlenecks within the MEP pathway of Z. mobilis using enzyme overexpression strains. Altogether, our data indicate that the reaction catalyzed by DXS represents the first metabolic bottleneck and rate-limiting enzyme in the Z. mobilis MEP pathway and that the overexpression of this enzyme is a viable approach to increase carbon flow into this pathway. These findings are consistent with observations in other bacteria. Previous studies in B. subtilis, E. coli, R. sphaeroides, and Corynebacterium glutamicum have shown that the overexpression of DXS alone or in combination with other MEP enzymes can enhance MEP pathway activity and improve downstream isoprenoid (i.e., isoprene or carotenoids) generation (20, 27, 30, 38, 39, 45–47).

The overexpression of DXS2 in Z. mobilis led to a large and rapid accumulation in MEcDP (Fig. 3A and Fig. 4A), suggesting a second major bottleneck at IspG and/or IspH. Previous research in plants and other bacteria have observed similar patterns in MEcDP accumulation resulting from DXS overexpression (29, 48) and have shown that the additional overexpression of IspG mitigates this bottleneck and improves downstream isoprenoid (i.e., lycopene) production (49). Consistent with these prior studies, the dual overexpression of DXS2 and IspG in Z. mobilis effectively reduced the accumulation of MEcDP and successfully mobilized carbon to the downstream metabolites HMBDP and IDP/DMADP (Fig. 4B). When we overexpressed DXS2 in combination with IspG and IspH, we observed only a minor accumulation in HMBDP (Fig. 4C), suggesting an effective mitigation of the bottleneck at IspH observed during DXS2 and IspG overexpression. While DXS2, IspG, and IspH overexpression still displayed an accumulation in MEcDP, the overall reduction in the combined pool of MEP intermediates suggests enhanced carbon flow through the MEP pathway in this strain. Consistent with DXS2 being the first rate-limiting step in the MEP pathway, the individual overexpression of IspG and IspH in Z. mobilis was insufficient to increase carbon flow into the MEP pathway (Fig. 3).

Although we measured robust protein overexpression for IspG and other MEP pathway enzymes, IspH overexpression was not as strong (Fig. S1). In line with these observations, a previous study tracked dynamic changes in IspG and IspH protein levels in Z. mobilis overexpression strains postinduction and found that while IspG protein levels increased by 38-fold (compared to wild-type Z. mobilis) by 120 min postinduction, IspH levels increased only by 4.6-fold (50). The reason for this disparity in protein overexpression between IspH and the other MEP enzymes in Z. mobilis is unknown and requires further investigation; however, it is possible that the iron-sulfur (4Fe-4S) cluster in the active site of IspH is less stable and more susceptible to oxidative damage than the one in IspG, which could lead to rapid turnover of IspH and a failure to attain high overexpression levels (9).

Similar to DXS2, our analyses revealed that the individual overexpression of the bifunctional enzyme IspDF in Z. mobilis can increase carbon flow into the MEP pathway (Fig. 3). However, when we overexpressed DXS2 and IspDF in combination, we observed two apparent bottlenecks at MEcDP and CDP-ME, with only a minor accumulation (2-fold) in IDP/DMADP (see Fig. S5 in the supplemental material). These results suggest that IspE could be a minor enzymatic constraint, but the large accumulation in MEcDP still supports the finding that during DXS2 overexpression in Z. mobilis, IspG and IspH become rate-limiting steps.

10.1128/msystems.00092-23.5 FIG S5 The combinatorial overexpression of DXS2 and IspDF causes accumulations in CDP-ME and MEcDP. Fold changes in MEP metabolites were measured relative to Z. mobilis overexpressing GFP. Data represent averages of three biological replicates. Error bars show ± standard deviation. Some error bars are too small to be visible in this representation. Asterisks indicate statistical significance (FDR, <0.05). Abbreviations: DXP, 1-deoxy-d-xylulose 5-phosphate; MEP, 2-C-methyl-d-erythritol 4-phosphate; CDP-ME, 4-diphosphocytidyl-2-C-methyl-d-erythritol; CDP-MEP, 4-diphosphocytidyl-2-C-methyl-d-erythritol 2-phosphate; MEcDP, 2-C-methyl-d-erythritol 2,4-cyclodiphosphate; HMBDP, 4-hydroxy-3-methylbut-2-enyl diphosphate; IDP, isopentenyl diphosphate; DMADP, dimethylallyl diphosphate; DXS2, DXP synthase; IspDF, bifunctional enzyme MEP cytidyl transferase/MEcDP synthase. Download FIG S5, PDF file, 0.1 MB. Copyright © 2023 Khana et al. 2023 Khana et al. https://creativecommons.org/licenses/by/4.0/ This content is distributed under the terms of the Creative Commons Attribution 4.0 International license.

Although the combined overexpression of DXS2 and IspS, resulted in measurable isoprene production, the additional overexpression of other MEP enzymes (i.e., IDI, IspG, and IspH) together with DXS2 and IspS did not further improve isoprene production (Table 2). While the reason for this finding is presently unclear and requires additional investigation, it is plausible that competing native isoprenoid synthesis pathways (i.e., ubiquinol, menaquinone, squalene, and hopanoids), which previous studies indicate are highly active in Z. mobilis (7, 26), siphon IDP and DMADP away from IspS. It is therefore possible that the reduction in MEP metabolite levels we observed in the strains overexpressing DXS2 and IspS in combination with other MEP pathway enzymes correlates with an increase in downstream isoprenoids we did not monitor and that increasing isoprene production beyond the levels we observed would require knocking out or downregulating these competing pathways. In addition, the DXS2_IspG_IspH_IspS and DXS2_IspG_IspH_IspS_IDI strains exhibited heavily reduced growth and glucose consumption rates (see Table S3 in the supplemental material), which could have also contributed to their reduced isoprene production.

10.1128/msystems.00092-23.9 TABLE S3 Growth and glucose consumption rates for Z. mobilis overexpression strains. Download Table S3, DOCX file, 0.02 MB. Copyright © 2023 Khana et al. 2023 Khana et al. https://creativecommons.org/licenses/by/4.0/ This content is distributed under the terms of the Creative Commons Attribution 4.0 International license.

Thermodynamics dictate reaction reversibility and enzyme efficiency in metabolic pathways (51–53). Although experimentally derived Gibbs free energies (Δ*G*) for MEP pathway reactions are unavailable, standard Δ*G* estimates for DXS, DXR, IspD, IspE, and IspH can be obtained computationally using the component contribution method (CCM; see Materials and Methods) (54). Standard Δ*G* values using the CCM could not be estimated for the IspF and IspG reactions due to the high uncertainty of the computationally predicted standard free energy of formation for MEcDP (54). To provide insight into the *in vivo* thermodynamics of the MEP pathway in Z. mobilis, we combined these computational standard Δ*G* estimates with our MEP pathway metabolite concentration data. We found that the MEP pathway in wild-type Z. mobilis appears to be highly thermodynamically favorable (see Table S4 in the supplemental material). The estimated *in vivo* Δ*G* values for DXS, DXR, and IspH were all highly energetically favorable, with Δ*G* values of <−29 kJ/mol, while IspD was slightly less favorable (Δ*G*, −12 kJ/mol). The large changes in MEP pathway metabolite concentrations that we observed throughout our overexpression strains did not result in thermodynamic bottlenecks, with the sole exception of the IspDF overexpression strain, which appeared to be thermodynamically constrained at IspD (ΔG, −2.7 kJ/mol) due to the large accumulation in CDP-ME (Fig. 3). Although standard Δ*G* estimates using the CCM could not be obtained for IspF, the natively high intracellular concentration of MEcDP in wild-type Z. mobilis and its significant accumulation postoverexpression of DXS2 suggest that this reaction must be highly thermodynamically favorable.

10.1128/msystems.00092-23.10 TABLE S4 Standard and *in vivo* Δ*G* values for MEP pathway reactions in wild-type Z. mobilis. Download Table S4, DOCX file, 0.02 MB. Copyright © 2023 Khana et al. 2023 Khana et al. https://creativecommons.org/licenses/by/4.0/ This content is distributed under the terms of the Creative Commons Attribution 4.0 International license.

Based on the findings from this study, future engineering efforts to improve isoprenoid production in Z. mobilis can focus on optimizing expression levels of rate-limiting enzymes. Tuning MEP pathway enzyme expression will be critical to maximize carbon flow through the pathway while minimizing the burden on the cell (45, 55, 56). Additionally, maximizing MEP pathway activity in Z. mobilis will likely involve increased activity/expression of auxiliary enzymes/proteins to the MEP pathway. Specifically, IspG and IspH contain [4Fe-4S] clusters, which are synthesized and assembled via the *suf* operon (9, 57, 58). IspG and IspH rely on accessory proteins to resupply electrons to the [4Fe-4S] clusters after each catalytic cycle (59, 60). In plants (*A. thaliana*) and bacteria (E. coli), electrons are resupplied to IspG and IspH [4Fe-4S] clusters via ferredoxin (FdxA) and flavodoxin I (FldA), respectively (61, 62). Oxidized FdxA/FldA proteins are subsequently reduced by flavodoxin/ferredoxin NADP^+^ reductase (Fpr), which uses NADPH as the preferred electron donor (59, 62, 63). Thus, in addition to overexpression, IspG and/or IspH activity in Z. mobilis could be enhanced by increasing [4Fe-4S] biogenesis activity via the overexpression of the *suf* operon, overexpressing electron transfer accessory proteins (i.e., FdxA, FldA, and/or Fpr) (64), and/or increasing the supply of NADPH (65, 66).

Moreover, future efforts to improve isoprenoid production in Z. mobilis will likely need to consider allosteric regulation or posttranslational modification of MEP pathway enzymes. Our time course experiments revealed complex dynamic changes in the levels of MEP pathway intermediates during enzyme overexpression (Fig. 4 and 5). Although the rapid increases in MEP pathway intermediates can be attributed to increased enzyme activity postinduction, it remains unknown as to why the levels of some of the MEP intermediates gradually decreased over time after their initial spike. It is plausible that feedback regulation may be responsible for these interesting dynamics. For example, previous research in plants has established that DXS is a major metabolic control point in the MEP pathway (29, 67) and that DXS is subject to feedback inhibition from IDP and DMADP (68). Additionally, an *in vitro* study showed that recombinant IspF from E. coli can be feedforward activated by MEP and that IspF is subject only to feedback inhibition from the downstream isoprenoid FPP when IspF is complexed with MEP (69).

The results of this study represent an initial step toward establishing Z. mobilis as a viable candidate for the industrial production of isoprenoids. By monitoring acute responses in metabolism to individual and combinatorial MEP enzyme overexpression, we revealed valuable insights into the bottlenecks of the Z. mobilis MEP pathway. Ultimately, these insights can be leveraged to inform metabolic engineering efforts in Z. mobilis to generate commodity molecules.

## MATERIALS AND METHODS

### Strains and growth conditions.

Wild-type and engineered strains of Z. mobilis ZM4 (ATCC 31821) were streaked onto *Zymomonas* rich-medium glucose (ZRMG) plates (10 g/L yeast extract, 2 g/L KH_2_PO_4_, 20 g/L glucose, and 20 g/L agar) from 25% glycerol stocks and incubated in an anaerobic (5% H_2_, 5% CO_2_, 90% N_2_ atmosphere, and <100 ppm O_2_) chamber (Coy Laboratory) at 30°C for 3 to 4 days. Single colonies were used to inoculate liquid ZRMG (10 g/L yeast extract, 2 g/L KH_2_PO_4_, and 20 g/L glucose) containing 100 μg/mL spectinomycin. Strains were grown overnight and then subcultured into 2 to 5 mL of *Zymomonas* minimal media (ZMM) [1 g/L K_2_HPO_4_, 1 g/L KH_2_PO_4_, 0.5 g/L NaCl, 1 g/L (NH_4_)_2_SO_4_, 0.2 g/L MgSO_4_·7H_2_O, 0.025 g/L Na_2_MoO_4_·2H_2_O, 0.025 g/L FeSO_4_·7H_2_O, 0.02 g/L CaCl_2_·2H_2_O, 1 mg/L calcium pantothenate, and 20 g/L glucose]. The subcultured growth was incubated overnight and used to inoculate experimental cultures. All experimental cultures were inoculated at an initial OD_600_ of 0.02 to 0.05, and all medium was kept anaerobic for a minimum of 16 h prior to inoculation. For the single time point and time course experiments, strains were grown in either 25 mL or 100 mL of ZMM in 125-mL or 500-mL Erlenmeyer flasks, respectively, enclosed with foil, and with a stir bar set at 120 rpm. For the single-time-point experiments, cells were grown to an OD_600_ of 0.2 before induction with 0.5 mM IPTG. Intracellular metabolites were collected for three biological replicates when cells reached an OD_600_ of 0.5. For the time course experiments, cells were grown to an OD_600_ of 0.35 before induction with 0.5 mM IPTG. Intracellular metabolites were collected for three biological replicates before induction (time point 0 min) and 7.5, 15, 30, 45, 60, and 120 min postinduction.

### Generation of plasmid constructs and engineered strains.

All genes were cloned into the plasmid pRL814 (Table S2), which was provided courtesy of Robert Landick (Professor, UW-Madison, Department of Biochemistry, Great Lakes Bioenergy Research Center). The pRL814 plasmid was generated by assembling a fragment derived from pIND4 (70) containing the *lacI_q_* gene, P_T7A1-O34_, and a pRH52 (13) fragment containing GFP, the pBBR-1 broad host origin of replication and *aadA* for spectinomycin resistance (32). PCR primers were designed using the New England BioLabs (NEB) assembly tool (https://nebuilderv1.neb.com/) to amplify gene fragments from template DNA (genomic or plasmid DNA) and to overlap the plasmid backbone. Synthetic ribosome binding sites (RBSs) of 20 to 30 bp in length were generated for each gene using the RBS library calculator (https://salislab.net/software/) (71, 72) and introduced into pRL814 via overlapping PCR primers. The plasmid constructs were assembled via Gibson assembly (73) using NEB HiFi DNA assembly master mix reagents. Each 25-μL Gibson assembly reaction contained 0.015 pmol total of linearized vector backbone DNA and 0.03 to 0.09 pmol total of gene fragment DNA, and reaction mixtures were incubated for 1 h at 50°C. Constructed plasmids were then introduced into E. coli DH5α cells via transformation and grown on LB plates (10 g/L tryptone, 5 g/L yeast extract, 5 g/L NaCl, and 15 g/L agar) with 100 μg/mL spectinomycin. Extracted plasmids from single colonies were screened via Sanger sequencing (Functional Biosciences) to confirm the successful transformation of the plasmid construct. The constructs were then introduced into Z. mobilis ZM4 triple (Δ*hsdS_c_*, Δ*mrr*, and Δ*cas3*) or quadruple (Δ*hsdS_c_*, Δ*hsdS_p_*, Δ*mrr*, and Δ*cas3*) mutant background strains using a previously described conjugation method (74–76). Successful conjugation into Z. mobilis was confirmed via PCR, and 25% glycerol stocks were prepared of the engineered strains.

### Intracellular metabolite extractions.

When bacterial cultures reached the appropriate OD_600_ (0.35 or 0.5), 10 mL of liquid culture was extracted in the anaerobic chamber using a serological pipette. Cells were separated from the media by vacuum filtering the culture through a 0.45-μm-pore-size hydrophilic nylon filter (Millipore; catalog no. HNWP04700) applied to a sintered glass funnel. The nylon filter containing cells was immediately immersed cell-side down into a plastic petri dish (5.5-cm diameter) containing 1.5 mL cold (–20°C) extraction solvent (40:40:20 by % volume methanol-acetonitrile-water; all high-performance liquid chromatography [HPLC] grade) and kept on a chilled aluminum block. This process simultaneously lysed the cells, quenched metabolism, and dissolved intracellular metabolites. The petri dish was lightly swirled to ensure complete contact of solvent with the filter. Filters remained in the cold solvent for ~15 min before being repeatedly rinsed in the extraction solvent to collect any remaining cell debris and metabolites (77–79). The cell-solvent mixture was then transferred to a prechilled 1.5-mL microcentrifuge tube, removed from the anaerobic chamber, and centrifuged at 16,000 × *g* for 10 min at 4°C, and the supernatant was collected for LC-MS analysis.

### Intracellular metabolite sample preparation for HPLC-MS.

For the single-time-point experiments, 180 μL of extraction solvent containing intracellular metabolites was dried under N_2_ gas and resuspended in 60 μL of solvent A (97:3 H_2_O:methanol with 10 mM tributylamine adjusted to pH 8.2 using 10 mM acetic acid). For the time course experiments, 90 μL of experimental sample was combined with 90 μL of intracellular metabolite extract (collected as described previously) from a reference sample of Z. mobilis ZM4 grown in ZMM containing universally labeled [U-^13^C] glucose (Cambridge Isotope Laboratories; item no. CLM-1396-PK) as the sole carbon source. The same reference sample was used for all time course samples in a given experiment to correct for LC-MS variation between sample injections. The combined sample was then dried under N_2_ gas and resuspended in 60 μL solvent A. Following resuspension, samples were briefly vortexed for 5 to 10 s and centrifuged at 16,000 × *g* for 10 min at 4°C to remove any remaining cell debris. The supernatant was then transferred to an HPLC vial for LC-MS analysis.

### Metabolomics LC-MS methods.

Metabolomics LC-MS analyses were conducted using a Vanquish ultra-high-performance liquid chromatography (UHPLC) system (Thermo Scientific) coupled to a hybrid quadrupole-Orbitrap mass spectrometer (Q Exactive; Thermo Scientific) equipped with electrospray ionization operating in negative-ion mode. The chromatography was performed at 25°C using a 2.1- by 100-mm reverse-phase C_18_ column with a 1.7-μm particle size (Water; Acquity UHPLC ethylene-bridged hybrid [BEH]). The chromatography gradient used solvent A and solvent B (100% methanol) and was as follows: 0 to 2.5 min, 5% B; 2.5 to 17 min, linear gradient from 5% B to 95% B; 17 to 19.5 min, 95% B; 19.5 to 20 min, linear gradient from 95% B to 5% B; and 20 to 25 min, 5% B. The flow rate was held constant at 0.2 mL/min. For the targeted metabolomics method, eluent from the column was injected into the MS for analysis until 18 min, at which point flow was redirected to waste for the remainder of the run. To increase the quality of signal for the MEP metabolites, an alternative method was employed in which only eluent from 7.5 to 18 min was injected into the MS, which permitted for higher injection volumes. The MS parameters included the following: full MS-single ion monitoring (SIM) scanning between 70 and 1,000 *m/z* and 160 and 815 *m/z* for the targeted metabolomics and MEP metabolite-specific methods, respectively; automatic gain control (AGC) target, 1e6; maximum injection time (IT), 40 ms; and a resolution of 70,000 full width at half maximum (FWHM).

### Quantification of intracellular metabolite concentrations.

Intracellular metabolite concentrations were measured by growing six biological replicates of Z. mobilis ZM4 in ZMM containing [U-^13^C] glucose as the sole carbon source and extracting intracellular metabolites using extraction solvent containing known concentrations of non-isotopically labeled standards. The solvent containing ^13^C-labeled intracellular metabolites and ^12^C-labeled standards was analyzed via LC-MS, and the ratio of labeled:unlabeled peak intensities was used to calculate the intracellular metabolite concentrations (31). Extracellular metabolites were collected and quantified to account for excreted pyruvate and MEcDP (9). At the time of extraction, 11 mL of bacterial culture was obtained via a serological pipette and centrifuged at 4,000 × *g* for 10 min at 4°C. The supernatant (10 mL) was then subjected to filter sterilization and metabolite extraction, as described previously. Due to their chemical similarity (i.e., similar structure and the same molecular formula), we were unable to separate the isomers IDP and DMADP via the nonchiral column that we used for our LC-MS analyses. Thus, these isomers were quantified as a combined pool.

### Aniline derivatization and LC-MS separation of IDP and DMADP.

To allow separate measurements of IDP and DMADP, we performed aniline derivatization (80) and used an alternative LC-MS method with a chiral column that allowed for the separation of pure standards of the isomers IDP and DMADP (Echelon Biosciences) and some cellular samples. Due to the larger particle size of the chiral column (5.0 μm), this method is less sensitive, and thus, the ability to separate IDP and DMADP was only successful in strains (i.e., DXS2_IspS) that produced high levels of these metabolites. Standards of IDP and DMADP were prepared at 35 μM in 100 μL of HPLC-grade H_2_O, and following intracellular metabolite extraction of cellular samples, 100 μL of extract was dried under N_2_ gas and resuspended in 100 μL of HPLC-grade H_2_O. Following the addition of 10 μL of a 200-mg/mL solution of *N*-(3-dimethylaminopropyl)-*N*-ethylcarbodiimide hydrochloride (EDC) and 10 μL of a 6 M aniline solution, samples were vortexed for 2 h. The derivatization was stopped via the addition of 5 μL of triethylamine. Standards/samples were centrifuged at 16,000 × *g* for 5 min at ambient temperature, and the supernatant was transferred to an HPLC vial for LC-MS analysis.

The chromatography to separate the isomers IDP and DMADP was performed at 35°C using a 4.6- by 250-mm β-cyclodextrin chiral column with a 5.0-μm particle size (Astec Cyclobond I 2000). The chromatography gradient used solvent 1 (50 mM ammonium acetate, pH 4.5) and solvent 2 (90:10 acetonitrile:H_2_O) and was as follows: 0 to 2 min, 100% B; 2 to 30 min, linear gradient from 100% B to 40% B; 30 to 45 min, linear gradient from 40% B to 25% B; 45 to 70 min, 25% B; 70 to 71 min, linear gradient from 25% B to 100% B; and 71 to 81 min, 100% B. The flow rate was held constant at 0.6 mL/min. The MS parameters used were as described previously with a scanning range of 150 to 600 *m/z*.

### Metabolomics computational analysis.

Data analysis was performed using the El-MAVEN (Elucidata) software (81). Compounds were identified based on retention times matched to pure standards. For the single-time-point experiments, fold changes in metabolite levels in the MEP enzyme overexpression strains were measured against Z. mobilis overexpressing GFP. Signal intensities were log_2_ transformed and two-tailed *t* tests were performed assuming equal variance to measure *P* values. For the time course experiments, the ^12^C parent signal (derived from the experimental sample) was divided by the fully labeled ^13^C signal (derived from the spiked-in ^13^C-labeled Z. mobilis reference sample) to normalize for LC-MS variation between injections. The ^12^C-to-^13^C ratio was then normalized to the OD_600_ at which metabolites were extracted, and fold changes were measured against the preinduction (time point 0) state. Signal intensities were log_2_ transformed, and paired two-tailed *t* tests were performed to measure *P* values. *P* values were then corrected for multiple-hypothesis testing using the Benjamini-Hochberg method using a false discovery rate (FDR) of 5% (82).

### Isoprene production measurements.

To measure isoprene production, at least three biological replicates of Z. mobilis strains overexpressing GFP and isoprene synthase were grown anaerobically as described previously. Cultures were induced with 0.5 mM IPTG at an OD_600_ of 0.35. At 3 h postinduction, 200 μL was transferred to a sealed vial and 2 mL was used for OD_600_ measurements. Following 10 min of additional growth, 1 mL of headspace was sampled via a needle and syringe and injected into a fast isoprene sensor (Hills-Scientific) to calculate isoprene concentrations (43). Two-tailed *t* tests comparing isoprene production in the GFP overexpression strain to the strains overexpressing IspS were performed assuming equal variance to measure *P* values.

Growth rates (h^−1^) and glucose consumption rates (mmol_glucose_ grams dry cell weight^−1^ · h^−1^) were obtained by anaerobically growing three biological replicates of Z. mobilis overexpression strains as described previously. OD_600_ readings and glucose measurements were collected every hour until stationary phase was reached (see Fig. S6 in the supplemental material). Glucose measurements were performed by taking supernatants from bacterial growth cultures and diluting them 1/50 with HPLC-grade H_2_O, mixed 50:50 with 1 mM [U-^13^C] glucose, and analyzed via LC-MS using a C_18_ column (as described previously). The chromatography gradient used solvent A and solvent B (100% methanol) and was as follows: 0 to 2.5 min, 5% B; 2.5 to 8 min, linear gradient from 5% B to 95% B; 8 to 10.5 min, 95% B; 10.5 to 11 min, linear gradient from 95% B to 5% B; and 11 to 15 min, 5% B. The MS parameters used were as described previously with a scanning range of 70 to 1,000 *m/z*. The ratio of labeled:unlabeled peak intensities was used to calculate glucose concentrations. Growth and glucose consumption rates were normalized to grams per dry cell weight (gDCW) (10).

10.1128/msystems.00092-23.6 FIG S6 Growth curves for Z. mobilis overexpression strains. Overexpression strains were grown anaerobically in ZMM (see Materials and Methods), and OD_600_ measurements were collected every hour. The red segments of each line graph represent the growth window at which metabolite extractions were performed. Data represent averages of three biological replicates per strain. Error bars show ± standard deviation. Some error bars are too small to be visible in this representation. Abbreviations: IspS, isoprene synthase; DXS2, DXP synthase; IDI, IDP isomerase; IspG, HMBDP synthase; IspH, HMBDP reductase. Download FIG S6, PDF file, 0.8 MB. Copyright © 2023 Khana et al. 2023 Khana et al. https://creativecommons.org/licenses/by/4.0/ This content is distributed under the terms of the Creative Commons Attribution 4.0 International license.

### Protein extraction and sample preparation for proteomics.

At the time of metabolite extraction, 10 mL of bacterial culture was collected for one or three biological replicates, and cells were centrifuged for 2.5 min at 4,000 × *g* at 4°C. The supernatant was discarded, and the cell pellets were flash frozen in liquid nitrogen and stored at −80°C until further analysis. To prepare the samples for proteomics analysis, cell pellets were thawed and lysed by resuspension in 6 M guanidine hydrochloride. The samples were subjected to three rounds of heating to 100°C for 5 min and re-equilibration to ambient temperature for 5 min. The total protein concentration was quantified using a Pierce bicinchoninic acid (BCA) protein assay kit (Thermo Scientific), and 50 to 100 μg of protein was used for further processing. Methanol was added to a final concentration of 90%, followed by centrifugation at 15,000 × *g* for 5 min. The supernatant was discarded, and the protein pellets were dried for 10 min. The pellets were then resuspended in 200 μL of lysis buffer [8 M urea, 100 mM Tris (pH 8.0), 10 mM tris(2-carboxyethyl)phosphine (TCEP) hydrochloride, and 40 mM chloroacetamide] to denature, reduce, and alkylate proteins. Resuspended proteins were diluted to 1.5 M urea in 100 mM Tris (pH 8.0). Trypsin was added at a ratio of 50:1 sample protein concentration to trypsin and incubated overnight (~12 h) at ambient temperature. The trypsinization reaction was stopped using 10% trifluoroacetic (TFA) acid. Following protein digestion, each sample was desalted using a Strata-X 33 μM polymeric reversed-phase styrene divinylbenzene solid-phase extraction cartridge and was dried. Before LC-MS/MS analysis, samples were reconstituted in 0.2% formic acid, and peptide concentrations were measured using a Pierce quantitative colorimetric peptide assay kit (Thermo Scientific).

### Proteomics LC-MS analysis.

For each analysis, 2 μg of peptides was loaded onto a 75-μm-inside-diameter (i.d.), 30-cm-long capillary with an imbedded electrospray emitter and packed in a 1.7-μm-particle-size C_18_ BEH column. The mobile phases used were as follows: phase A, 0.2% formic acid; and phase B, 0.2% formic acid–70% acetonitrile. Peptides were eluted with a gradient increasing from 0% to 75% B over 42 min followed by a 4-min 100% B wash and 10 min of equilibration in 100% A for a complete gradient of 60 min.

The eluting peptides were analyzed with an Orbitrap Fusion Lumos (Thermo Scientific) mass spectrometer. Survey scans were performed at a resolution of 240,000 with an isolation analysis at 300 to 1,350 *m/z* and AGC target of 1e6. Data-dependent top-speed (1-s) tandem MS/MS sampling of peptide precursors was enabled with dynamic exclusion set to 10 s on precursors with charge states 2 to 4. MS/MS sampling was performed with 0.7-Da quadrupole isolation and fragmentation by higher-energy collisional dissociation (HCD) with a collisional energy value of 25%. The mass analysis was performed in the ion trap using the “turbo” scan speed for a mass range of 200 to 1,200 *m/z*. The maximum injection time was set to 11 ms, and the AGC target was set to 20,000.

### Proteomics computational analysis.

Raw LC-MS files were analyzed using the MaxQuant software (version 1.5.8.3) (83). Spectra were searched using the Andromeda search engine against a target decoy database. Label-free quantitation and match between runs were toggled on, MS/MS tolerance was set to 0.4 Da, and the number of measurements for each protein was set to 1. Default values were used for all other analysis parameters. The peptides were grouped into subsumable protein groups and filtered to reach 1% false discovery rate (FDR) based on the target decoy approach. Log_2_-transformed label-free quantitation intensities were further processed to obtain log_2_ fold change values relative to Z. mobilis overexpressing GFP or background signal determined by randomly generated signal derived from a distribution in the noise range.

### Thermodynamic estimates of MEP pathway reactions.

Standard free energy estimates were obtained using the component contribution method (CCM) via the Python package equilibrator-api (version 0.4.7) (57). The following chemical parameters were used for our free energy estimates: pMg, 3: ionic strength, 0.25 M; temperature, 30°C; and pH 6 (84). Standard free energy estimates using the CCM were unavailable for the MEP reactions IspF and IspG due to the high uncertainty of the computationally predicted standard free energy of formation for MEcDP. For the DXS reaction, the aqueous CO_2_ concentration was estimated based on Henry’s law and the gaseous CO_2_ concentration in the anaerobic chamber. For the IspD reaction, an intracellular estimate of 100 μM was used for diphosphate.
