# Supplementary material for: Systematic Analysis of Metabolic Bottlenecks in the Methylerythritol 4-Phosphate (MEP) Pathway of Zymomonas mobilis
Source: mSystems. 2023 Mar 30;8(2):e00092-23. doi: 10.1128/msystems.00092-23 (PMC10134818; doi:10.1128/msystems.00092-23)
Supplement: TABLE S1 [file msystems.00092-23-s0007.docx]

Table S1
Intracellular metabolite concentrations in *Z. mobilis* ZM4 with 95% confidence intervals

| **Metabolite** | **Abbreviation** | **Molarity (M)** | **95LB (M)** | **95UB (M)** | **Source** |
| --- | --- | --- | --- | --- | --- |
| Glucose 6-phosphate | G6P | 5.00E-03 | 3.94E-03 | 6.34E-03 | ■ |
| 6-phosphogluconate | 6PG | 2.09E-03 | 1.68E-03 | 2.60E-03 | ■ |
| 2-keto-3-deoxy-6-phosphogluconate | KDPG | 6.39E-03 | 5.34E-03 | 7.64E-03 | ■ |
| Glyceraldehyde 3-phosphate | GAP | 1.00E-03 | 5.06E-04 | 1.50E-03 | ▲ |
| Pyruvate | PYR | 6.35E-03 | 4.93E-03 | 7.76E-03 | ▲ |
| 3-phosphoglycerate | 3PG | 3.85E-03 | 3.36E-03 | 4.41E-03 | ■ |
| Phosphoenolpyruvate | PEP | 4.02E-05 | 3.56E-05 | 4.55E-05 | ■ |
| 1-deoxy-D-xylulose 5-phosphate | DXP | 1.68E-04 | 1.52E-04 | 1.84E-04 | ▲ |
| 2-C-methyl-D-erythritol 4-phosphate | MEP | 1.40E-05 | 1.30E-05 | 1.50E-05 | ▲ |
| 4-diphosphocytidyl-2-C-methyl-D-erythritol | CDP-ME | 3.60E-05 | 2.77E-05 | 4.42E-05 | ▲ |
| 2-C-methyl-D-erythritol 2,4-cylcodiphosphate | MEcDP | 1.25E-04 | 9.10E-05 | 1.59E-04 | ▲ |
| 4-hydroxy-3-methylbut-2-enyl-diphosphate | HMBDP | 7.40E-06 | 5.83E-06 | 8.98E-06 | ▲ |
| Isopentenyl diphosphate/ Dimethylallyl diphosphate | IDP/ DMADP | 7.09E-06 | 6.22E-06 | 7.96E-06 | ▲ |
| Geranyl pyrophosphate | GPP | 3.28E-06 | 2.87E-06 | 3.69E-06 | ▲ |
| Farnesyl pyrophosphate | FPP | 2.56E-05 | 2.30E-05 | 2.81E-05 | ▲ |
| Adenosine triphosphate | ATP | 2.81E-03 | 2.50E-03 | 3.13E-03 | ▲ |
| Adenosine diphosphate | ADP | 1.27E-03 | 1.21E-03 | 1.33E-03 | ▲ |
| Adenosine monophosphate | AMP | 8.89E-05 | 6.72E-05 | 1.18E-04 | ■ |
| Guanosine triphosphate | GTP | 1.72E-03 | 1.36E-03 | 2.17E-03 | ■ |
| Guanosine diphosphate | GDP | 4.23E-04 | 2.74E-04 | 6.52E-04 | ■ |
| Guanosine monophosphate | GMP | 5.16E-05 | 2.43E-05 | 1.10E-04 | ■ |
| Uridine triphosphate | UTP | 1.81E-03 | 1.38E-03 | 2.39E-03 | ■ |
| Uridine diphosphate | UDP | 4.47E-04 | 2.87E-04 | 6.97E-04 | ■ |
| Uridine monophosphate | UMP | 8.94E-05 | 7.34E-05 | 1.09E-04 | ■ |
| Cytidine triphosphate | CTP | 1.12E-03 | 9.86E-04 | 1.25E-03 | ▲ |
| Cytidine monophosphate | CMP | 3.42E-05 | 3.15E-05 | 3.70E-05 | ▲ |
| Nicotinamide adenine dinucleotide (reduced) | NADH | 4.13E-04 | 1.58E-04 | 1.08E-03 | ■ |
| Nicotinamide adenine dinucleotide (oxidized) | NAD^+^ | 2.21E-03 | 1.46E-03 | 3.33E-03 | ■ |
| Nicotinamide adenine dinucleotide phosphate (reduced) | NADPH | 5.51E-04 | 3.64E-04 | 8.33E-04 | ■ |
| Nicotinamide adenine dinucleotide phosphate (oxidized) | NADP^+^ | 3.00E-04 | 1.93E-04 | 4.66E-04 | ■ |

95% confidence intervals (LB95=lower bound, UB95=upper bound)

■^1^

▲This study

1. Jacobson, T. B. *et al.* 2H and 13C metabolic flux analysis elucidates in vivo thermodynamics of the ED pathway in Zymomonas mobilis. *Metab. Eng.* **54**, 301–316 (2019).
