# Supplementary figures and images for: Systematic Analysis of Metabolic Bottlenecks in the Methylerythritol 4-Phosphate (MEP) Pathway of Zymomonas mobilis
Source: mSystems. 2023 Mar 30;8(2):e00092-23. doi: 10.1128/msystems.00092-23 (PMC10134818; doi:10.1128/msystems.00092-23)

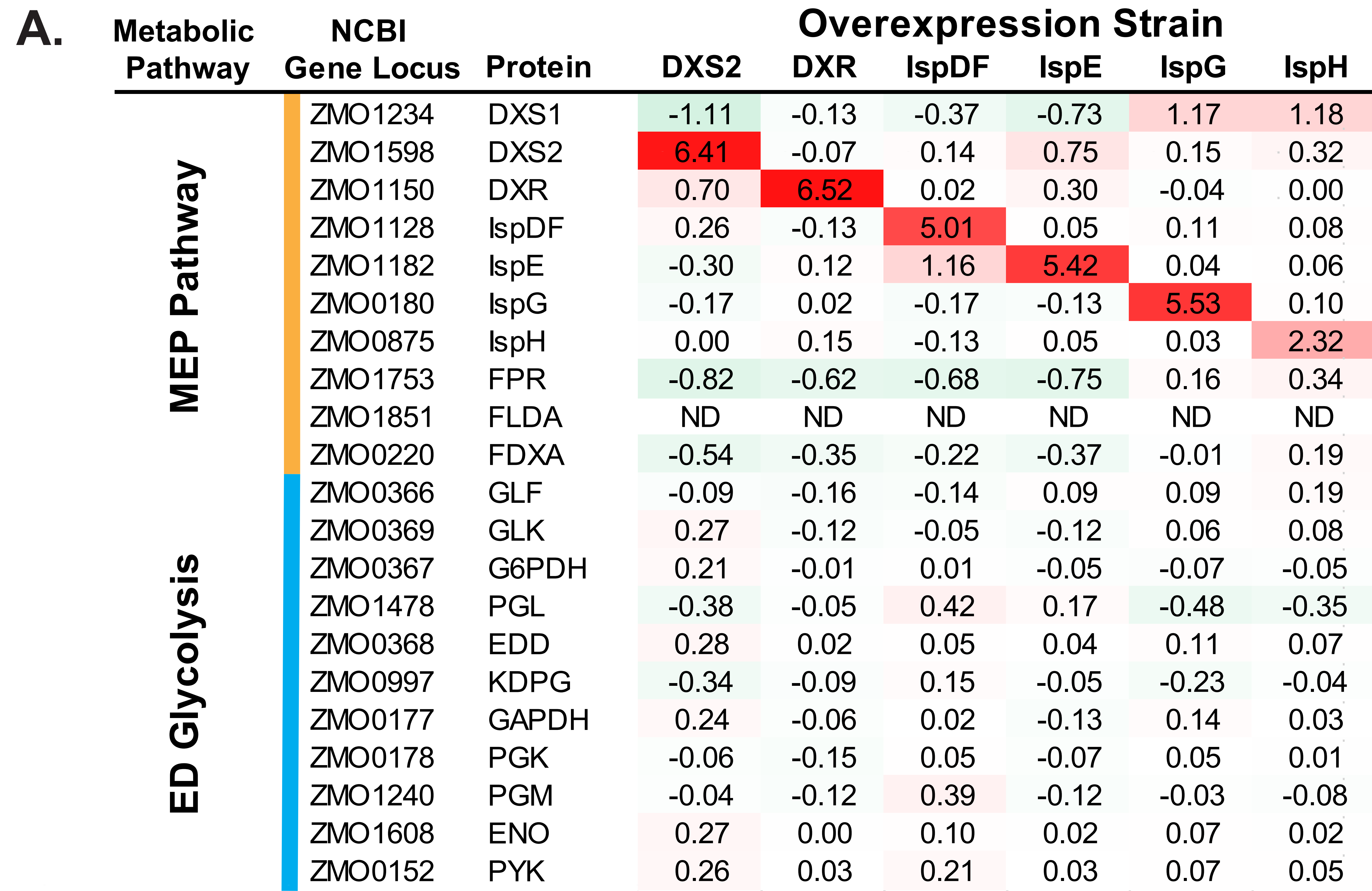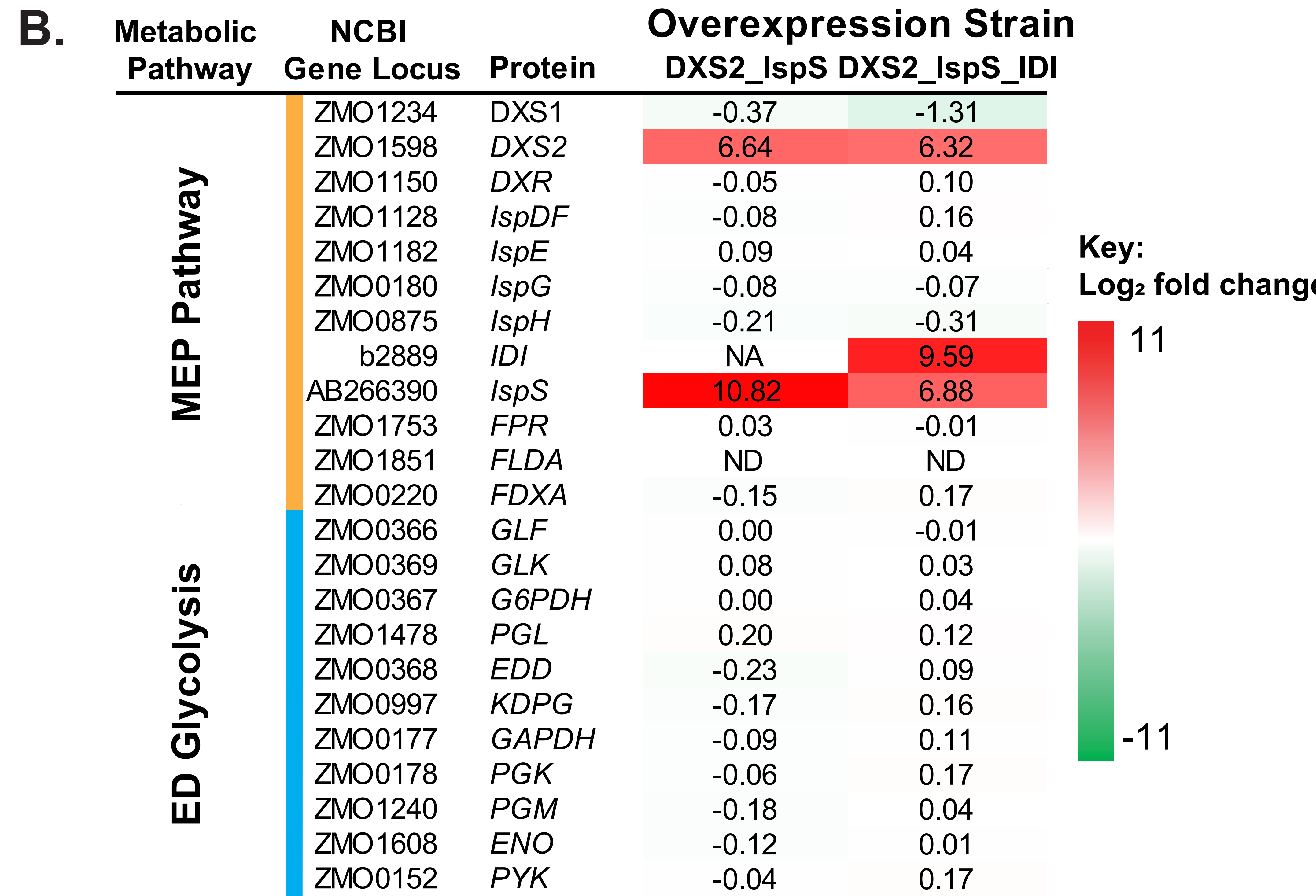

Supplement: FIG S1 [file msystems.00092-23-s0001.pdf]

A.

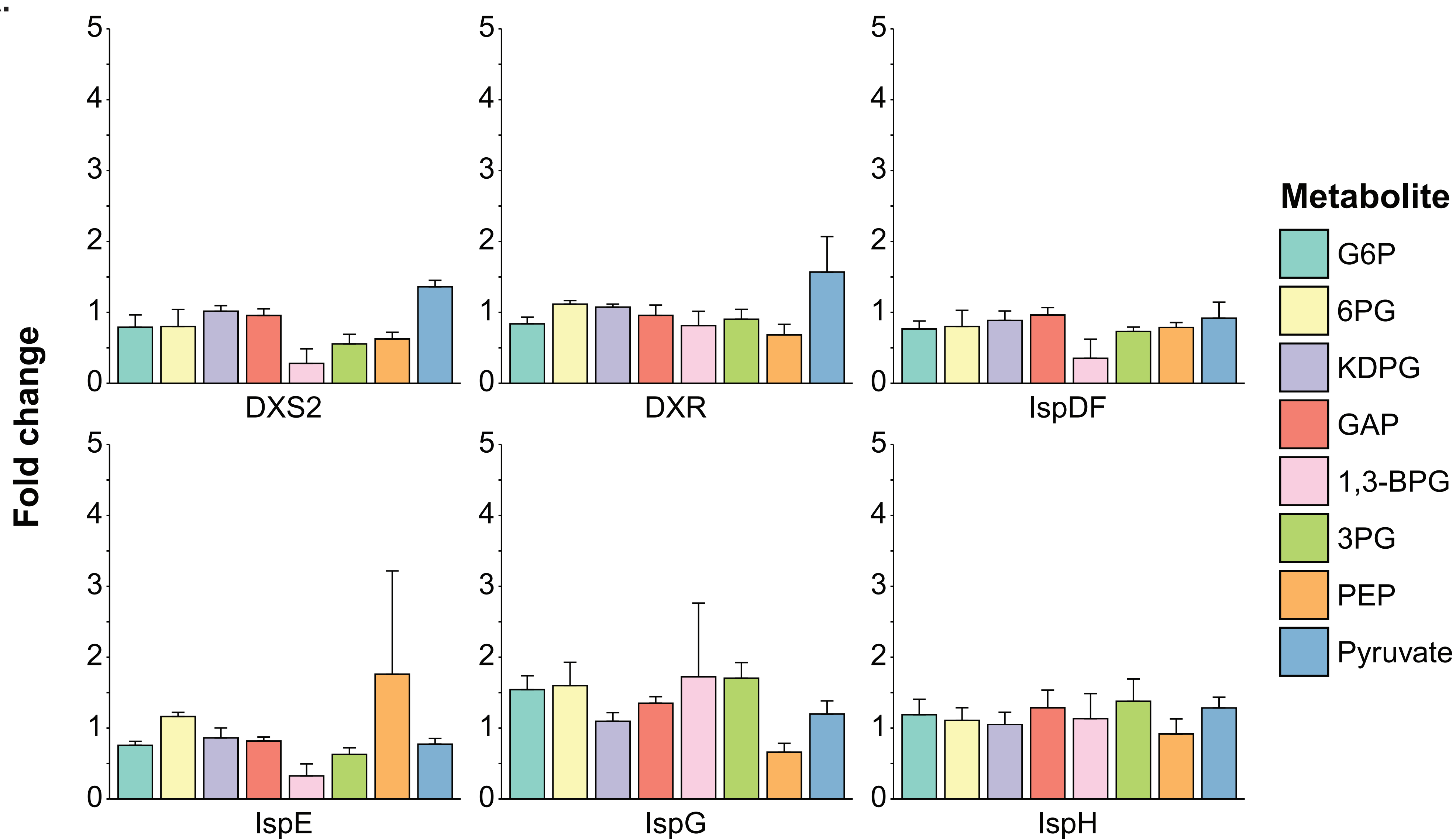

B.

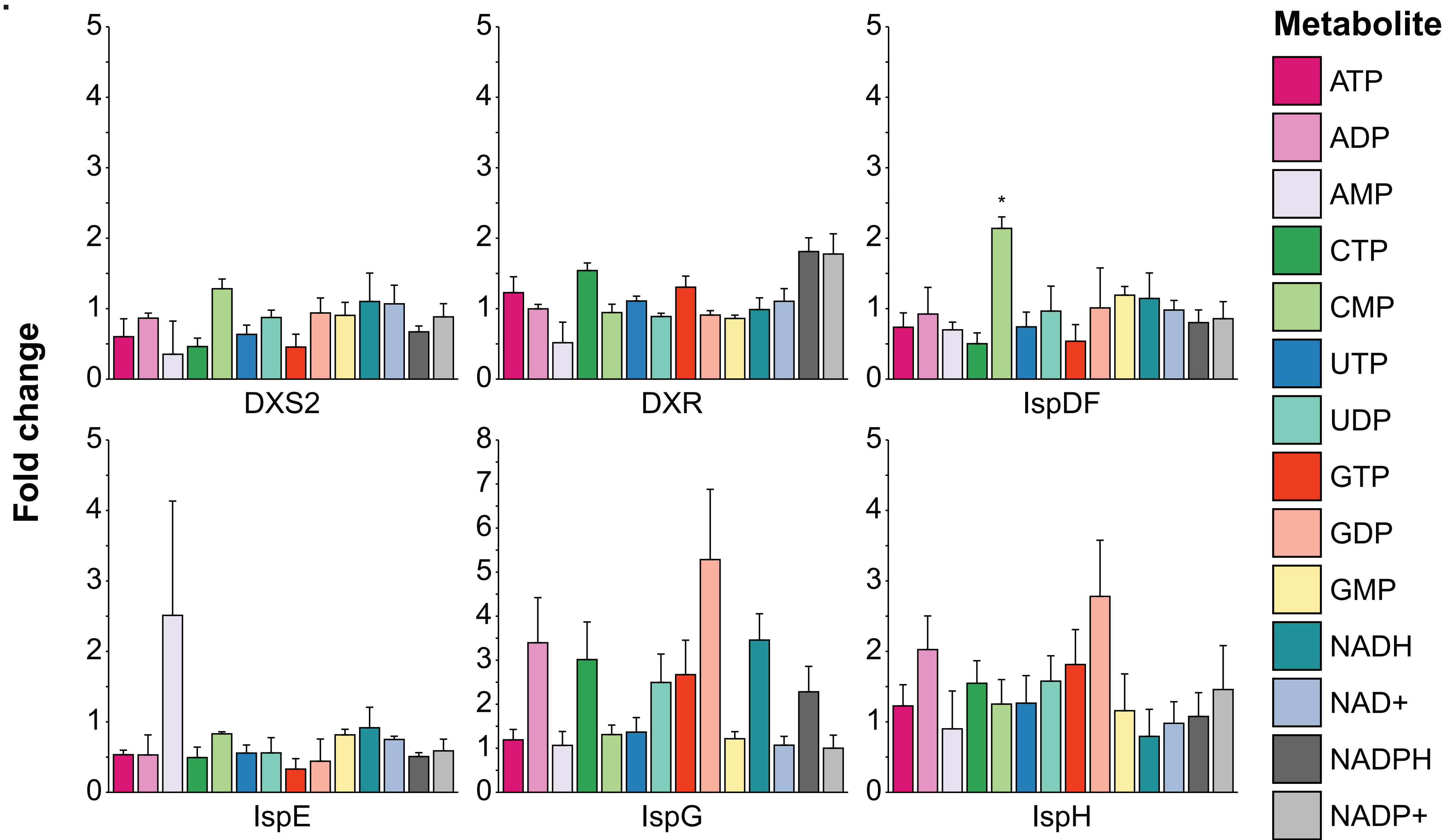

Supplement: FIG S2 [file msystems.00092-23-s0002.pdf]

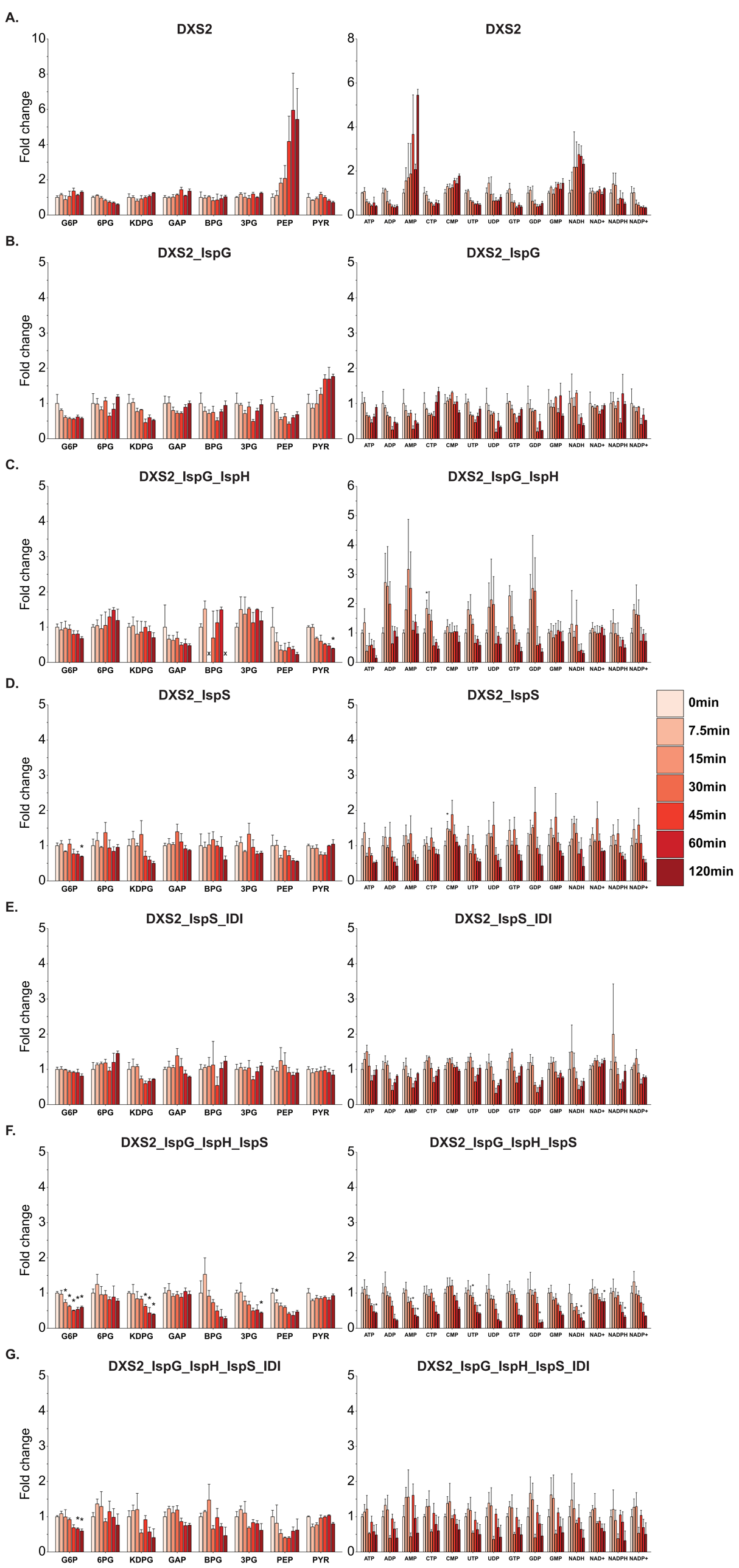

Supplement: FIG S3 [file msystems.00092-23-s0003.pdf]

**A.**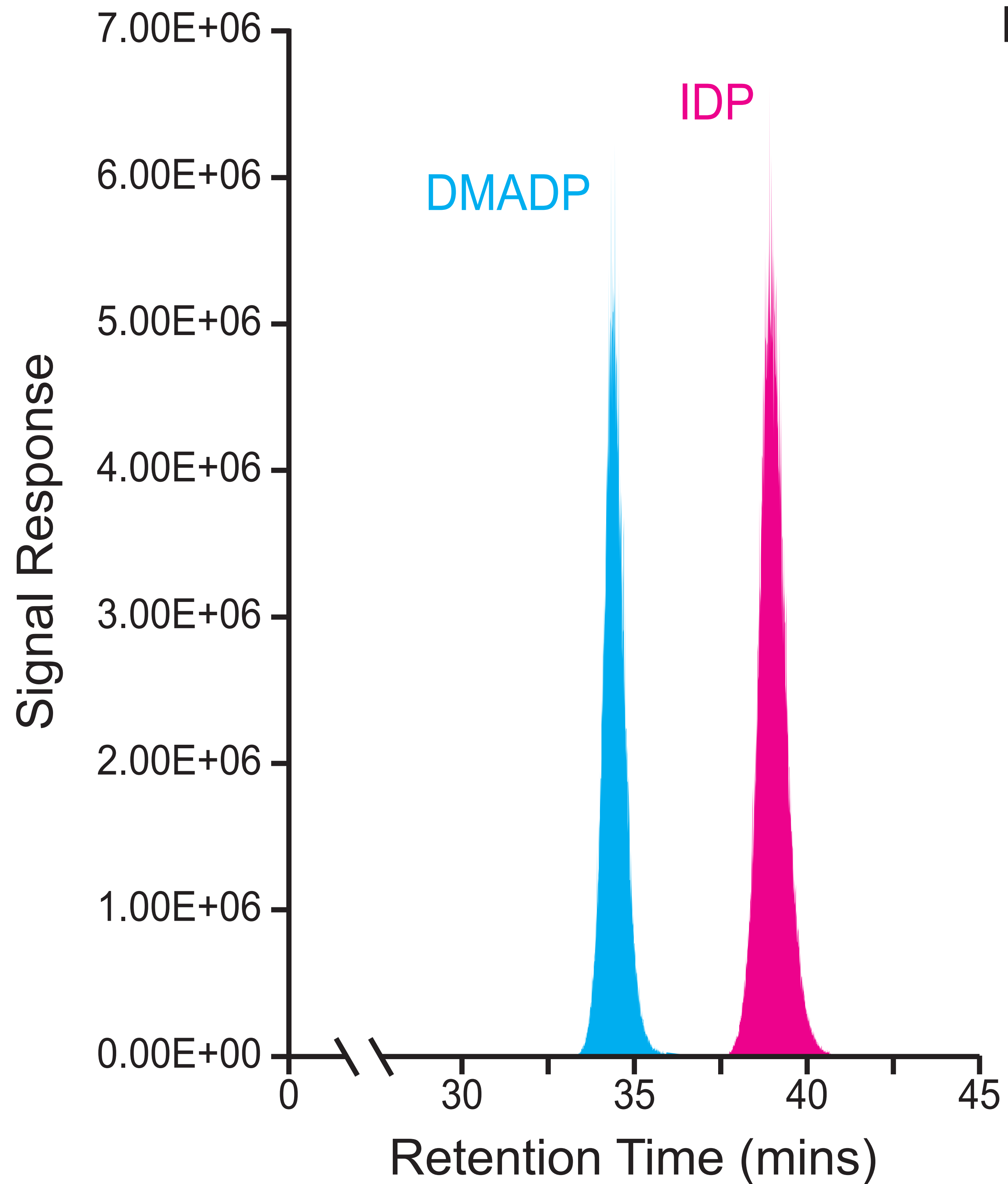**B.**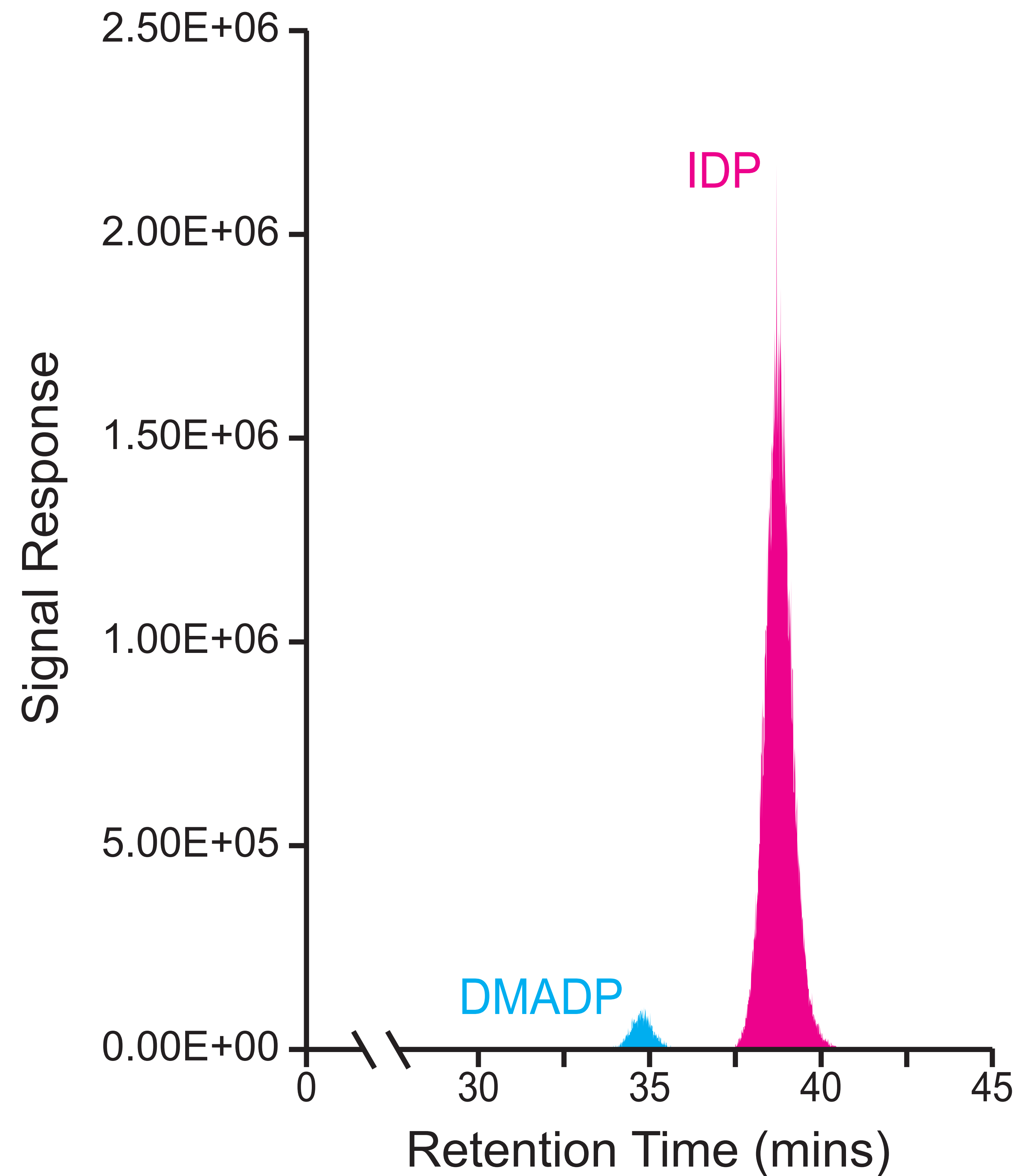

Supplement: FIG S4 [file msystems.00092-23-s0004.pdf]

**Fold change**

100  
90  
80  
70  
60  
50  
40  
30  
20  
10  
0

**DXS2\_IspDF**

**MEP Metabolite**

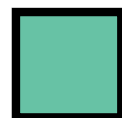

DXP

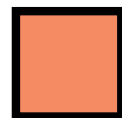

MEP

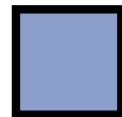

CDP-ME

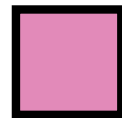

CDP-MEP

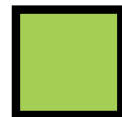

MEcDP

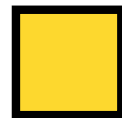

HMBDP

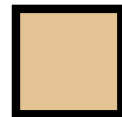

IDP/ DMADP

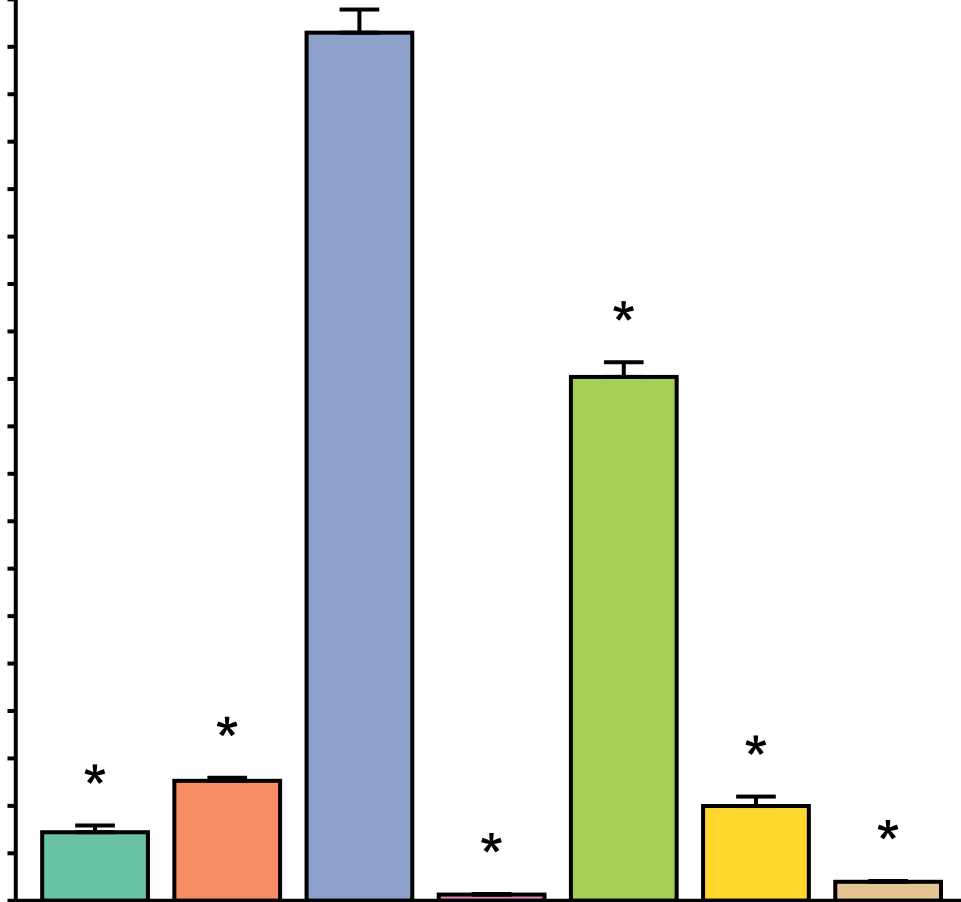

Supplement: FIG S5 [file msystems.00092-23-s0005.pdf]

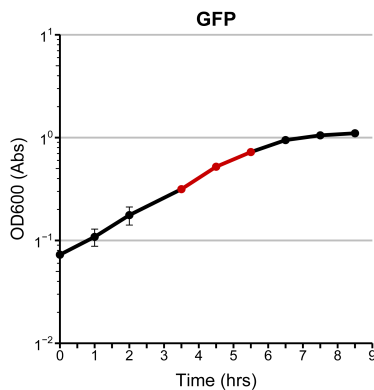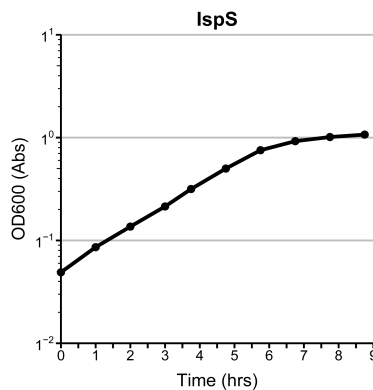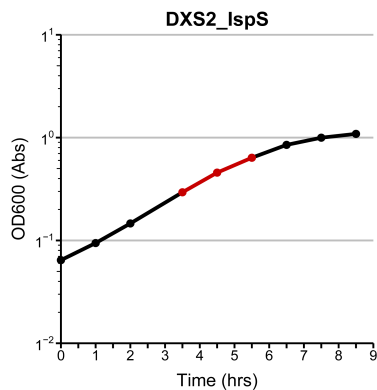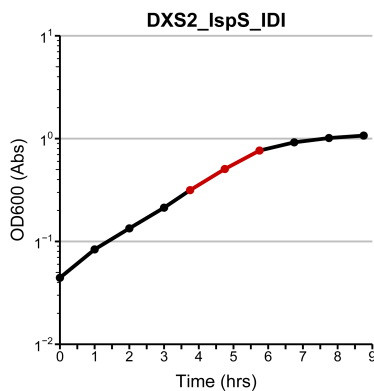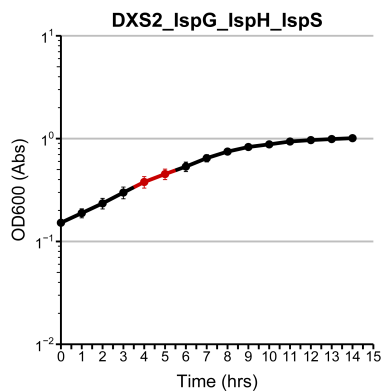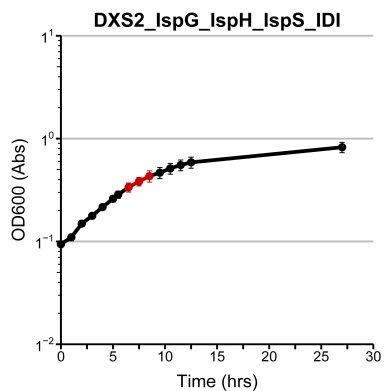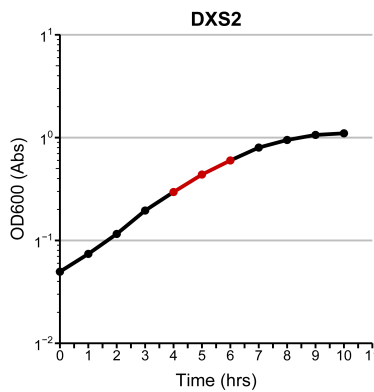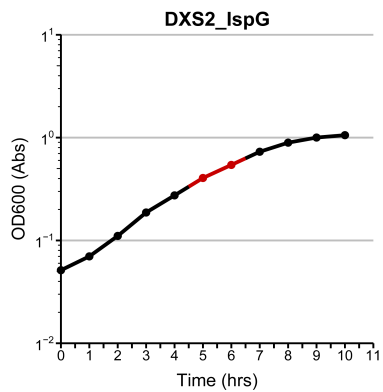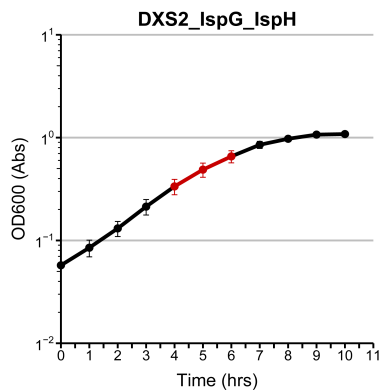

Supplement: FIG S6 [file msystems.00092-23-s0006.pdf]
