# Supplementary material for: Systematic Analysis of Metabolic Bottlenecks in the Methylerythritol 4-Phosphate (MEP) Pathway of Zymomonas mobilis
Source: mSystems. 2023 Mar 30;8(2):e00092-23. doi: 10.1128/msystems.00092-23 (PMC10134818; doi:10.1128/msystems.00092-23)
Supplement: TABLE S2 [file msystems.00092-23-s0008.docx]

Table S2

List of engineered *Z. mobilis* strains constructed for this study

| **Strain Name** | **Genes (Source)** | **Background Strain^1^** |
| --- | --- | --- |
| ZM4_GFP | *GFP* (*A. victoria*) | ∆*hsdS_c_*, ∆*mrr*, ∆*cas3* |
| ZM4_DXS2 | *DXS2* (*Z. mobilis* ZM4) | ∆*hsdS_c_*, ∆*mrr*, ∆*cas3* |
| ZM4_DXR | *DXR* (*Z. mobilis* ZM4) | ∆*hsdS_c_*, ∆*mrr*, ∆*cas3* |
| ZM4_IspDF | *IspDF* (*Z. mobilis* ZM4) | ∆*hsdS_c_*, ∆*mrr*, ∆*cas3* |
| ZM4_IspE | *IspE* (*Z. mobilis* ZM4) | ∆*hsdS_c_*, ∆*mrr*, ∆*cas3* |
| ZM4_IspG | *IspG* (*Z. mobilis* ZM4) | ∆*hsdS_c_*, ∆*mrr*, ∆*cas3* |
| ZM4_IspH | *IspH* (*Z. mobilis* ZM4) | ∆*hsdS_c_*, ∆*hsdS_p_*, ∆*mrr*, ∆*cas3* |
| ZM4_DXS2_IspG | *DXS2*, *IspG* (*Z. mobilis* ZM4) | ∆*hsdS_c_*, ∆*mrr*, ∆*cas3* |
| ZM4_DXS2_IspG_IspH | *DXS2*, *IspG*, *IspH* (*Z. mobilis* ZM4) | ∆*hsdS_c_*, ∆*hsdS_p_*, ∆*mrr*, ∆*cas3* |
| ZM4_IspS | *IspS* (*E. globulus*) | ∆*hsdS_c_*, ∆*mrr*, ∆*cas3* |
| ZM4_DXS2_IspS | *DXS2* (*Z. mobilis* ZM4),  *IspS* (*E. globulus*) | ∆*hsdS_c_*, ∆*mrr*, ∆*cas3* |
| ZM4_DXS2_IspG_IspH_IspS | *DXS2*, *IspG*, *IspH* (*Z. mobilis* ZM4),  *IspS* (*E. globulus*), | ∆*hsdS_c_*, ∆*hsdS_p_*, ∆*mrr*, ∆*cas3* |
| ZM4_DXS2_IspS_IDI | *DXS2* (*Z. mobilis* ZM4),  *IspS* (*E. globulus*),  *IDI* (*E. coli* RL3000) | ∆*hsdS_c_*, ∆*mrr*, ∆*cas3* |
| ZM4_DXS2_IspG_IspH_IspS_IDI | *DXS2*, *IspG*, *IspH* (*Z. mobilis* ZM4),  *IspS* (*E. globulus*),  *IDI* (*E. coli* RL3000) | ∆*hsdS_c_*, ∆*hsdS_p_*, ∆*mrr*, ∆*cas3* |

1. Lal, P. B. *et al.* Improving Mobilization of Foreign DNA into Zymomonas mobilis Strain ZM4 by Removal of Multiple Restriction Systems. *Appl. Environ. Microbiol.* **87**, 1–16 (2021).
