# Supplementary material for: Systematic Analysis of Metabolic Bottlenecks in the Methylerythritol 4-Phosphate (MEP) Pathway of Zymomonas mobilis
Source: mSystems. 2023 Mar 30;8(2):e00092-23. doi: 10.1128/msystems.00092-23 (PMC10134818; doi:10.1128/msystems.00092-23)
Supplement: TABLE S3 [file msystems.00092-23-s0009.docx]

Table S3
Growth and glucose consumption rates for *Z. mobilis* overexpression strains

| **Strain Name** | **Overexpressing Genes** | **Growth Rate (h^-1^)^a^** | **Glucose Consumption Rate (mmol_glucose_ gDCW^-1^ h^-1^)^a^** |
| --- | --- | --- | --- |
| ZM4_GFP | GFP | 0.36 ± 0.005 | 64.9 ± 3.0 |
| ZM4_IspS | IspS | 0.36 ± 0.004 | 72.4 ± 2.6 |
| ZM4_DXS2_IspS | DXS2, IspS | 0.35 ± 0.001 | 57.3 ± 3.5 |
| ZM4_DXS2_IspS_IDI | DXS2, IspS, IDI | 0.36 ± 0.004 | 68.3 ± 6.9 |
| ZM4_DXS2_IspG_IspH_IspS | DXS2, IspG, IspH, IspS | 0.17 ± 0.012 | 33.8 ± 1.0 |
| ZM4_DXS2_IspG_IspH_IspS_IDI | DXS2, IspG, IspH, IspS, IDI | 0.14 ± 0.010 | 33.6 ± 4.9 |
| ZM4_DXS2 | DXS2 | 0.38 ± 0.001 | 71.3 ± 5.4 |
| ZM4_DXS2_IspG | DXS2, IspG | 0.36 ± 0.002 | 58.2 ± 4.8 |
| ZM4_DXS2_IspG_IspH | DXS2, IspG, IspH | 0.38 ± 0.007 | 74.6 ± 9.3 |

^a^Growth and glucose consumption rates were measured post enzyme overexpression and represent the average of three biological replicates per strain ± standard error.
