# Supplementary material for: Systematic Analysis of Metabolic Bottlenecks in the Methylerythritol 4-Phosphate (MEP) Pathway of Zymomonas mobilis
Source: mSystems. 2023 Mar 30;8(2):e00092-23. doi: 10.1128/msystems.00092-23 (PMC10134818; doi:10.1128/msystems.00092-23)
Supplement: TABLE S4 [file msystems.00092-23-s0010.docx]

Table S4
Standard and *in vivo* ∆G values for MEP pathway reactions in wild-type *Z. mobilis*

| **MEP Reaction** | **Standard** ∆G (kJ/mol) ^a^ | ***In vivo*** ∆G (kJ/mol) |
| --- | --- | --- |
| DXS | -37.3 | -45.1 |
| DXR | -21.5 | -29.3 |
| IspD | -8.3 | -12.0 |
| IspE | -4 | NA |
| IspF | NA | NA |
| IspG | NA | NA |
| IspH | -127.1 | -89.1 |

^a^Standard ∆G values were estimated using the Component Contribution Method (see Materials and Methods)

NA indicates unavailable ∆G values due to lack of metabolite concentration data and/ or standard ∆G estimates
